# Supplementary material for: Allosteric regulation and crystallographic fragment screening of SARS-CoV-2 NSP15 endoribonuclease
Source: Nucleic Acids Res. 2023 Apr 28;51(10):5255–70. doi: 10.1093/nar/gkad314 (PMC10250223; doi:10.1093/nar/gkad314)
Supplement: gkad314_Supplemental_Files [file gkad314_supplemental_files.zip › Supplementary datasets.zip!/188/Refine_9/01-BUSTER/Cycle-2/LIST.html]

Refining

**BUSTER 2.X**: A suite of programs for macromolecular structure refinement by maximum-likelihood and Bayesian methods  
Copyright © 1992-2020 Global Phasing Ltd.  
All rights reserved.  
Authors: G. Bricogne, E. Blanc, M. Brandl, C. Flensburg, P. Keller, W. Paciorek, P. Roversi, O. Smart, C. Vonrhein, T. Womack  

Please reference use of the software as:

- BUSTER-TNT 2.X, Global Phasing Ltd, Sheraton House, Cambridge CB3 0AX, UK

Version: 2.10.3 for linux64-ifort made on May 20 2020 at 01:14:34  
Licenced to: Diamond Light Source, Scientific Software  

```
---


### Parsing of the BUSTER cardfile

TITLE : autoBUSTER refinement

   Cell information

   Symmetry information

 Using CCP4 X-ray form factors.

   Missing structure chemical composition

 Using ATOM block for chemical composition.


   Shell information

### Parsing of the TNT cardfile


---


### Reading observations file

 Number of reflexions in input:  47008

      Number of accepted reflexions:  40456

      Number of rejected reflexions:      0

   Reflexions in test set:            2022

   Reflexions in working set:        38434


---


### 1. Generating initial model

 2.10 * Shannon sampling rate. NX NY NZ:   288  288  192


  Effective Shannon rates are:    2.44    2.44    2.23


   Allocated BUFFER of size:     61.172 Mb.

   Initial Geometry Information


 No missing atoms declared


   Summary of model volumes

 No missing atoms declared: no prior computed


 Computing bulk solvent model scattering


 NCSMASK PDB radius for solvent mask:     2.15 Angstrom

 running PDBSET (setting cell and spacegroup P1) ... done
 running NCSMASK [1] (creating mask, SMOOTH 1.14073) ... done
 running MAPDUMP (to check if reduction to unit cell is needed) ... done
 running MAPMASK (converting mask to map) ... done

   Generation of babslv by theta-filter

   Electron density in frg mask:  0.420 Å-1

 FOBS  put on rough absolute scale by Wilson plot :
           K        =  0.69780E+02
           B        =    -10.259

 

### Cycle 0001: MaxLik scaling and refinement


   MaxLik Scaling Cycle 0001

   Wilson plot

   Statistics in resolution bins just after scaling
```

|  |  |
| --- | --- |
|  | - Rxpct factors plot- Rxpct factors table- <Log-Lik Gain>- Ln Sigma A- Correlation Coefficients |

```
   <Log-Likelihood> of null-hypothesis

Structure Factor Amplitudes


   Average Figure of Merit

 Switching off refinement of overall temperature factor


 Added B =     0.7248 Å2 to all atoms in fragment


 Added B =     0.7248 Å2 to B_SOLV

```
gelly refinement module
 Authors (2004-2009) O. S. Smart, C. Flensburg, W. Paciorek, C. Vonrhein, 
                     M. Brandl, T. O. Womack and G. Bricogne
         (2010-2011) O. S. Smart, C. Flensburg, W. Paciorek, C. Vonrhein, 
                     T. O. Womack and G. Bricogne
         (2012-2014) O. S. Smart, C. Flensburg, W. Paciorek, C. Vonrhein, 
                     and G. Bricogne
         (2015-2020) G. Bricogne, C. Flensburg, W. Paciorek, C. Vonrhein
 Copyright © 2004-2020 Global Phasing Ltd. 
 All rights reserved.
 gelly module compilation date May 20 2020 01:11:20
 
 gelly uses routines from TNT geometry. References: 
     Tronrud, D. E., Ten Eyck, L. F., Matthews, B. W. (1987)
     "An Efficient General-Purpose Least-Squares Refinement Program 
     for Macromolecular Structures". Acta Crystallogr A, 43:489-501.
     Tronrud, D. E. (1997). "The TNT Refinement Package", 
     in Macromolecular Crystallography, Part B, Eds Charlie Carter 
     and Robert Sweet, Methods in Enzymology, 277:306-319.
 wildcard matching function by Julian Robichaux http://www.nsftools.com

 No -random_seed flag found. Random number stream initialised using:
2147483562
2147483398

 Control flags set to:  -autoncs -hydrogens_input_occ2zero \
                     -hydrogens_output_occ2heavy -screen \
                     100 -screen_sigma \
                     3.0 -keeppdb \
                     1000000 -taomonitor_verbose \
                     2 
 

 ############### Starting setup via TNT routines

 Setting up tnt routines including license check:

                   TNT Stereochemistry Program
 Version: 5.22.3.0
 Licence for Diamond Light Source, Scientific Software is valid
 
 Using TNT geometry routines to load the tnt control file: gelly.tnt
 Using TNT routines to load initial coordinates from file fragment.cor
 Expecting 6519 atoms

 Found     2194 "NOTE BUSTER_RESIDUE_ATOM_DATA" cards
 Have copied information for    6519 atoms from TNT to gelly's store 
 
 The TNT WEIGHT cards used by this run:
 -----WEIGHT       BOND     2.0
 -----WEIGHT       ANGLE    2.0
 -----WEIGHT       IMPROPER 0.0
 -----WEIGHT       TORSION  2.0
 -----WEIGHT       PSEUDO   0.0
 -----WEIGHT       TRIGONAL 2.0
 -----WEIGHT       PLANE    5.0
 -----WEIGHT       CONTACT  5.0
 -----WEIGHT       BCORREL  10.0
 -----WEIGHT       CHIRAL   5.0
 -----WEIGHT       NCS      50.0
 -----WEIGHT       IDEAL    4.0
 -----WEIGHT       RFACTOR  9.01
 
 Dictionary versions picked up from NOTE BUSTER_RESTRAINT_DICT cards as: 
 ------ protgeo_eh99.dat (V1.6.4.17) 20191001  STANDARD AMINO ACID DICTIONARY. BONDS AND ANGLES FROM ENGH AND HUBER EH99. OTHER VALUES BASED ON PREVIOUS TNT OR TAKEN FROM CCP4. INCLUDES HYDROGEN ATOMS.
 ------ exoticaa.dat (V1.3.4.15) 20200327  COLLECTION OF NON-STANDARD AMINO ACIDS, MAINLY EH91 WITHOUT IDEAL DISTANCE INFO
 ------ nuclgeo.dat (V1.13.4.8) 20190909
 ------ bcorrel.dat (V1.15.10.2) 20190616
 ------ contact.dat (V1.15.12.10) 20191121
 ------ idealdist_contact.dat (V1.3.4.12) 20191001  IDEAL-DISTANCE CONTACT TERM DATA AS USED IN PROLSQ. VALUES USED HERE ARE BASED ON DATA HARVESTING OF PDB AS OF 20190109.
 ------ restraints for LIG (Unknown) from cif dictionary Z1262327505.cif
 ------ restraints for CIT (CITRIC ACID) from cif dictionary CIT.cif; generated by GRADE_PDB_LIGAND 1.2.12 (October 4 2016) using MOGUL 1.7.2(RC1), CSD as538be, with quantum mechanics RM1 ((DatabaseID PDB-CIT))
 ------ assume.dat (V1.9.4.2) 20180705
 
 Have picked up spacegroup name for pdb output as 'P 63      '
 Setting up Chain/Residue arrays
 
 Have command line specification -autoncs*
     Have found       1 NCS relations
     Pairwise matching residue number and type between chains: 
                    A        B        S        W    
          A         .      100%       -        -   
          B                  .        -        -   
          S                           .        -   
          W                                    .   
       Table key--                                                       
          all   : two chains have 100% match and same number of residues 
         multi  : ditto and they contribute to a multi chain ncs cluster 
          ***%  : two chains have >80% matching residue number/type      
           -    : chains appear to be unrelated                          
      chain A     have identified ncs to     1 other chains. So will weight each autoncs by (       6.666/#)=      6.6660
      chain B     have identified ncs to     1 other chains. So will weight each autoncs by (       6.666/#)=      6.6660
      chain S     have identified ncs to     0 other chains. 
      chain W     have identified ncs to     0 other chains. 
     For .Gelly cards produced see below - after pruning
 
 Setting up BUSTER_SET groups
 Have found    89 BUSTER_SET cards
 Now silently applying automatic/dictionary set definitions. For a verbose listing use '-verbose_set' flag.
 Applying user defined set definitions:
 =====NOTE BUSTER_SET ncsautoXcld = Water --> set 'ncsautoXcld' now has   966 atoms 
 =====NOTE BUSTER_SET ncsautoABset = Chain_A \ ncsautoXcld --> set 'ncsautoABset' now has  2783 atoms 
 Have defined    49 sets: 
      set:                 'All' has   6519 atoms in   1667 residues - automatically defined
      set:               'Empty' has      0 atoms in      0 residues - automatically defined
      set:            'ExoticAA' has      0 atoms in      0 residues - defined by dictionary file
      set:           'ExoticNUC' has      0 atoms in      0 residues - defined by dictionary file
      set:              'NotHET' has   5514 atoms in    698 residues - defined by dictionary file
      set:             'Chain_A' has   2783 atoms in    351 residues - automatically defined
      set:             'Chain_B' has   2770 atoms in    350 residues - automatically defined
      set:             'Chain_S' has    567 atoms in    567 residues - automatically defined
      set:             'Chain_W' has    399 atoms in    399 residues - automatically defined
      set:               'Water' has    966 atoms in    966 residues - defined by dictionary file
      set:          'StdProtein' has   5514 atoms in    698 residues - defined by dictionary file
      set:             'Protein' has   5514 atoms in    698 residues - defined by dictionary file
      set:                'Back' has   2794 atoms in    698 residues - defined by dictionary file
      set:                'Side' has   2720 atoms in    650 residues - defined by dictionary file
      set:        'RnaCanonical' has      0 atoms in      0 residues - defined by dictionary file
      set:        'DnaCanonical' has      0 atoms in      0 residues - defined by dictionary file
      set:     'RnaDnaCanonical' has      0 atoms in      0 residues - defined by dictionary file
      set:     'DnaRnaCanonical' has      0 atoms in      0 residues - defined by dictionary file
      set:         'RnaExtended' has      0 atoms in      0 residues - defined by dictionary file
      set:         'DnaExtended' has      0 atoms in      0 residues - defined by dictionary file
      set:      'RnaDnaExtended' has      0 atoms in      0 residues - defined by dictionary file
      set:      'DnaRnaExtended' has      0 atoms in      0 residues - defined by dictionary file
      set:                 'Dna' has      0 atoms in      0 residues - defined by dictionary file
      set:                 'Rna' has      0 atoms in      0 residues - defined by dictionary file
      set:              'RnaDna' has      0 atoms in      0 residues - defined by dictionary file
      set:              'DnaRna' has      0 atoms in      0 residues - defined by dictionary file
      set:             'Nucleic' has      0 atoms in      0 residues - defined by dictionary file
      set:             'Polymer' has   5514 atoms in    698 residues - defined by dictionary file
      set: 'BufferWeichenberger' has     26 atoms in      2 residues - defined by dictionary file
      set:    'BufferAdditional' has      0 atoms in      0 residues - defined by dictionary file
      set:              'Buffer' has     26 atoms in      2 residues - defined by dictionary file
      set:         'MetalAlkali' has      0 atoms in      0 residues - defined by dictionary file
      set:  'MetalAlkalineEarth' has      0 atoms in      0 residues - defined by dictionary file
      set:     'MetalTransition' has      0 atoms in      0 residues - defined by dictionary file
      set: 'MetalPostTransition' has      0 atoms in      0 residues - defined by dictionary file
      set:    'MetalLanthanides' has      0 atoms in      0 residues - defined by dictionary file
      set:               'Metal' has      0 atoms in      0 residues - defined by dictionary file
      set:         'SugarAlphaD' has      0 atoms in      0 residues - defined by dictionary file
      set:         'SugarAlphaL' has      0 atoms in      0 residues - defined by dictionary file
      set:               'Sugar' has      0 atoms in      0 residues - defined by dictionary file
      set:               'Other' has     39 atoms in      3 residues - defined by dictionary file
      set:            'Hydrogen' has      0 atoms in      0 residues - defined by dictionary file
      set:         'nonHydrogen' has   6519 atoms in   1667 residues - defined by dictionary file
      set:             'OccZero' has      0 atoms in      0 residues - defined by dictionary file
      set:          'OccNonZero' has   6519 atoms in   1667 residues - defined by dictionary file
      set:             'OccFull' has   6474 atoms in   1664 residues - defined by dictionary file
      set:          'OccNonFull' has     45 atoms in      3 residues - defined by dictionary file
      set:         'ncsautoXcld' has    966 atoms in    966 residues - user specified
      set:        'ncsautoABset' has   2783 atoms in    351 residues - user specified
 No command line arguments '-occ_allhet' or '-occ_restyp' so will not setup automated occcupancy refinement
 
 Interpreting CONSTANT and FREE cards: 
 =====CONSTANT     OCC
 Have found and interpreted      1 CONSTANT cards in the TNT input. 
 Before any COMBINE - Number of atoms with fixed XYZ =     0
 Before any COMBINE - Number of atoms with fixed B   =     0
 Before any COMBINE - Number of atoms with fixed OCC =  6519
 Before any COMBINE - Number of atoms with fixed BIJ =   966
                            cf total number of atoms =  6519
 
 There are no COMBINE cards to be found in the TNT card input 
 
 There are no NOTE BUSTER_COMBINE_RES cards to be found in the TNT card input 

 ############### Working out full connectivity list


 ############### Working out equivalent atoms


 Residue with most atoms given below:
        A|58:N(TRP)         
        A|58:CA(TRP)        
        A|58:CB(TRP)        
        A|58:CG(TRP)        
        A|58:CD1(TRP)       
        A|58:NE1(TRP)       
        A|58:CE2(TRP)       
        A|58:CD2(TRP)       
        A|58:CE3(TRP)       
        A|58:CZ3(TRP)       
        A|58:CH2(TRP)       
        A|58:CZ2(TRP)       
        A|58:C(TRP)         
        A|58:O(TRP)         


 Have no '-equiv_check_plus' argument - so will take atom type into
 account when checking for equivalence of otherwise identical atoms.
 

 ############### Looking at dihedrals


 ############### Looking at chirals

 NOTE BUSTER_CHIRAL_ANGLECUT card found - read value=    -31.00 degs from it
 NOTE BUSTER_CHIRAL_SIGMA card found - read value=      1.70 degs from it
 RANGE allowed for B variables in refinement:   from      3.00000 to    300.00000

 ############### Preparing for PDB output


 ############### Processing extra restraints explicitly defined

 No 'NOTE BUSTER_TARGET' cards or -target?? args found (used to read coordinates files for soft ncs to external)
 No 'NOTE BUSTER_OCCSUM'     cards found - so not using sum of occupancy restraints
 No 'NOTE BUSTER_DISTANCE'   cards found - so not setting up any additional atom-atom distance restraints
 No 'NOTE BUSTER_UTILANGLE'  cards found - so not setting up any additional restraints on any angle
 No 'NOTE BUSTER_UTILTOR'    cards found - so not setting up any additional sinusoidal restraints on torsions
 
 --- Have some similarity definitions 
     Similarity restraints on RMSD use superposition routines from: 
     Coutsias, E.A., Seok, C., Dill, K.A.(2004)
     "Using quaternions to calculate RMSD", J. Comput. Chem., 25:1849-1857 

 Have found    1 'NOTE BUSTER_SIM_DEFINE' cards 
     Maximum number of chains involved in any SIM group (excluding the template)=     1


 ########## Interpreting card: NOTE BUSTER_SIM_DEFINE ncsautoAB ncsautoABset B
     Number of atoms in template set=  2783
     Number of related chains (other than template) in SIM set=    1
     List of chains to be restrained (excluding template)=   B   
     Pairwise RMSD, XYZ in Angstroms, for SIM group: ncsautoAB
                 A         B   
     A         -----     ----- 
     B         0.692     ----- 
     Overall RMSD,XYZ for the SIM group is     0.692 Angstroms
     Pairwise rms deviation of [individual B's minus their set/chain average] in Angs**2
                 A         B         Bavg
     A         -----     -----    118.76
     B         48.35     -----     82.37
     Overall RMSD,B   for the SIM group is    48.354 Angs**2
     The number of common atoms for the above fits:
                 A         B   
     A         -----     ----- 
     B          2770     ----- 
     (compared to the template set that has    2783 atoms in it)
     List of atoms that are absent in one or more chains for this group: 
                                    A    B   
                    C01     501     +    -
                    N02     501     +    -
                    C03     501     +    -
                    C04     501     +    -
                    C05     501     +    -
                    C06     501     +    -
                    C07     501     +    -
                    N08     501     +    -
                    C09     501     +    -
                    C10     501     +    -
                    C11     501     +    -
                    N12     501     +    -
                    C13     501     +    -
     (key  +  atom present for this chain 
           -  atom absent  for this chain)
 transforms for each of the superpositions orthogonal matrix r11 r12 r13 r21 r22 r23 r31 r32 r33 tx ty tz (PDBSET syntax): 
TRANS_AB -0.874316 -0.484578 -0.027491 -0.484558  0.874724 -0.007805  0.027829  0.006497 -0.999592    33.980    33.061    14.666
 reverse transforms 
TRANS_BA -0.874316 -0.484558  0.027829 -0.484578  0.874724  0.006497 -0.027491 -0.007805 -0.999592    45.321   -12.549    15.852
 
 Setting up LSSR interatomic distance restraints
 Interpreting card: NOTE BUSTER_SIM_RESTRAIN_LSSR ncsautoAB 6.666
        setting up interatomic distance pair restraints with:
        Dmin=         0.00
        Dmax=         5.50
        sigma=        0.20
        Vmax=         3.00
        weight=       6.67
        number of distance pairs defined for this set is      34951
 Have not supplied either '-sim_swap_equiv' or '-sim_swap_equiv_plus' argument 
   so will not try swaping equivalent atoms to improve NCS and/or target agreement.
 
 NCS by LSSR outlier analysis
 -autoncs option automatic NCS prune routine -------- 
 Residues pruned out by gradient condition:
           A|54     A|131     A|135     A|140     A|147     A|148     A|150
          A|151     A|152     A|158     A|168     A|169     A|172     A|173
          A|174     A|175     A|181     A|182     A|184     A|185     A|186
          A|187     A|189     A|216     A|217     A|218     A|219     A|220
          A|221     A|222     A|237     A|250     A|253     A|255     A|256
          A|259     A|260     A|264     A|310     A|319     A|320     A|325
          A|326     A|327     A|328     A|329     A|344     A|345      B|54
          B|131     B|135     B|140     B|147     B|148     B|150     B|151
          B|152     B|158     B|168     B|172     B|173     B|174     B|175
          B|181     B|182     B|184     B|185     B|186     B|187     B|189
          B|216     B|217     B|218     B|219     B|220     B|221     B|222
          B|237     B|250     B|253     B|255     B|256     B|259     B|260
          B|264     B|310     B|319     B|320     B|325     B|326     B|327
          B|328     B|329     B|344     B|345
  
 Residues pruned out by function/max condition:
          A|149     A|168!    A|169!    A|170     A|171     A|172!    A|173!
          A|174!    A|187!    A|188     A|220!    A|259!    A|264!    A|326!
          A|346    A|ENDA     A|401     B|149     B|168!    B|169     B|170 
          B|171     B|172!    B|173!    B|174!    B|187!    B|188     B|220!
          B|259!    B|264!    B|326!    B|346    B|ENDA     B|401 
  
 Total number of residues for which LSSR restraints have been pruned out is      110 out of      700 participating
 Number of LSSR restraints pruned out is      7326 this is   21.0% of the total           34951
  
 -autoncs .Gelly cards suitable for adaptation for manual definition/pruning
           (note auto pruning is done in a slightly different way so exact results may differ slightly)
 -autoncs option .Gelly cards also written to file  auto_gelly_cards.txt
NOTE BUSTER_SET ncsautoXcld = Water    # card added - produced by -autoncs
NOTE BUSTER_SET ncsautoXcld = ncsautoXcld + { A|54 A|131 A|135 A|140 A|147 }    # card added - produced by -autoncs
NOTE BUSTER_SET ncsautoXcld = ncsautoXcld + { A|148 A|149 A|150 A|151 A|152 }    # card added - produced by -autoncs
NOTE BUSTER_SET ncsautoXcld = ncsautoXcld + { A|158 A|168 A|169 A|170 A|171 }    # card added - produced by -autoncs
NOTE BUSTER_SET ncsautoXcld = ncsautoXcld + { A|172 A|173 A|174 A|175 A|181 }    # card added - produced by -autoncs
NOTE BUSTER_SET ncsautoXcld = ncsautoXcld + { A|182 A|184 A|185 A|186 A|187 }    # card added - produced by -autoncs
NOTE BUSTER_SET ncsautoXcld = ncsautoXcld + { A|188 A|189 A|216 A|217 A|218 }    # card added - produced by -autoncs
NOTE BUSTER_SET ncsautoXcld = ncsautoXcld + { A|219 A|220 A|221 A|222 A|237 }    # card added - produced by -autoncs
NOTE BUSTER_SET ncsautoXcld = ncsautoXcld + { A|250 A|253 A|255 A|256 A|259 }    # card added - produced by -autoncs
NOTE BUSTER_SET ncsautoXcld = ncsautoXcld + { A|260 A|264 A|310 A|319 A|320 }    # card added - produced by -autoncs
NOTE BUSTER_SET ncsautoXcld = ncsautoXcld + { A|325 A|326 A|327 A|328 A|329 }    # card added - produced by -autoncs
NOTE BUSTER_SET ncsautoXcld = ncsautoXcld + { A|344 A|345 A|346 A|ENDA A|401 }    # card added - produced by -autoncs
NOTE BUSTER_SET ncsautoABset = Chain_A \ ncsautoXcld    # card added - produced by -autoncs
NOTE BUSTER_SIM_DEFINE ncsautoAB ncsautoABset B    # card added - produced by -autoncs
NOTE BUSTER_SIM_RESTRAIN_LSSR ncsautoAB 6.666    # card added - produced by -autoncs

 LSSR function contribution before pruning     51524.4
                             after pruning      6314.4
 Graph of which residues pruned from LSSR: ncs_pruned.mtv
 Graph of LSSR function outliers ncs_function_outliers.mtv
 Graph of LSSR gradient outliers ncs_gradient_outliers.mtv
 LSSR - analysis of distance difference distribution for NCS
         DeltaD less than 0.5 Angs:  proportion of population= 99.22% average DeltaD of subset     0.012 Angs
 Histogram of LSSR deltaD distribution ncs_histogram.mtv
 
 Interpreting card: NOTE BUSTER_TLS_SET tls1 { A|* }
 Interpreting card: NOTE BUSTER_TLS_SET tls2 { B|* }
 Interpreting card: NOTE BUSTER_TLS_SET tls1 { A|* }
 Interpreting card: NOTE BUSTER_TLS_T   tls1     -2.34221E-01 -1.01327E-01  3.03964E-01 -4.69383E-02  3.04493E-02 -1.84208E-02
 Interpreting card: NOTE BUSTER_TLS_L   tls1      1.34657E+00  1.70940E+00  9.47056E-01 -1.47356E-01 -1.08231E+00  4.33010E-01
 Interpreting card: NOTE BUSTER_TLS_S   tls1      1.90995E-01 -3.20182E-01  2.99606E-01 -2.04932E-01  7.24381E-02 -2.58989E-01 -5.15178E-02 -2.36847E-01
 Interpreting card: NOTE BUSTER_TLS_O   tls1     -2.31731E+01 -8.40215E+01  1.10770E+01
 Interpreting card: NOTE BUSTER_TLS_SET tls2 { B|* }
 Interpreting card: NOTE BUSTER_TLS_T   tls2     -2.94169E-01 -2.16545E-01  3.03964E-01  2.06527E-02  3.69318E-02 -2.62022E-02
 Interpreting card: NOTE BUSTER_TLS_L   tls2      1.48295E+00  1.25715E+00  8.55781E-01  5.77497E-01 -7.83848E-01 -7.57220E-01
 Interpreting card: NOTE BUSTER_TLS_S   tls2      1.16036E-02  8.23206E-02 -1.48910E-01  5.36303E-02  1.62509E-01  2.17161E-02 -7.83320E-02  1.02175E-01
 Interpreting card: NOTE BUSTER_TLS_O   tls2      2.22040E+01 -7.22942E+01  3.58164E+00
 Interpreting card: NOTE BUSTER_TLS_FIX tls1 ALL
 Interpreting card: NOTE BUSTER_TLS_FIX tls2 ALL


 TLS information. Total number of groups:     2


 Group   Natoms    FixRB   FixTLS   Group Tag    Group Spec
     1     2783      Y        Y     tls1    { A|* }
     2     2770      Y        Y     tls2    { B|* }
 
NOTE BUSTER_TLS_SET tls1 { A|* }
NOTE BUSTER_TLS_T   tls1     -2.34221E-01 -1.01327E-01  3.03964E-01 -4.69383E-02  3.04493E-02 -1.84208E-02
NOTE BUSTER_TLS_L   tls1      1.34657E+00  1.70940E+00  9.47056E-01 -1.47356E-01 -1.08231E+00  4.33010E-01
NOTE BUSTER_TLS_S   tls1      1.90995E-01 -3.20182E-01  2.99606E-01 -2.04932E-01  7.24381E-02 -2.58989E-01 -5.15178E-02 -2.36847E-01
NOTE BUSTER_TLS_O   tls1     -2.31731E+01 -8.40215E+01  1.10770E+01
NOTE BUSTER_TLS_FIX tls1 ALL
 
NOTE BUSTER_TLS_SET tls2 { B|* }
NOTE BUSTER_TLS_T   tls2     -2.94169E-01 -2.16545E-01  3.03964E-01  2.06527E-02  3.69318E-02 -2.62022E-02
NOTE BUSTER_TLS_L   tls2      1.48295E+00  1.25715E+00  8.55781E-01  5.77497E-01 -7.83848E-01 -7.57220E-01
NOTE BUSTER_TLS_S   tls2      1.16036E-02  8.23206E-02 -1.48910E-01  5.36303E-02  1.62509E-01  2.17161E-02 -7.83320E-02  1.02175E-01
NOTE BUSTER_TLS_O   tls2      2.22040E+01 -7.22942E+01  3.58164E+00
NOTE BUSTER_TLS_FIX tls2 ALL


 TLS: mmCIF group specification begin


#
loop_
_pdbx_refine_tls_group.id
_pdbx_refine_tls_group.refine_tls_id
_pdbx_refine_tls_group.beg_PDB_ins_code
_pdbx_refine_tls_group.beg_auth_asym_id
_pdbx_refine_tls_group.beg_auth_seq_id
_pdbx_refine_tls_group.end_PDB_ins_code
_pdbx_refine_tls_group.end_auth_asym_id
_pdbx_refine_tls_group.end_auth_seq_id
_pdbx_refine_tls_group.selection
_pdbx_refine_tls_group.selection_details
   1   1 . A   -1 . A  346 . '{ A|* }'
   2   1 . A  401 . A  501 . '{ A|* }'
   3   2 . B   -1 . B  346 . '{ B|* }'
   4   2 . B  401 . B  401 . '{ B|* }'
#


 TLS: mmCIF group specification end


 Interpreting card: NOTE BUSTER_ANISO_SET Empty
 Interpreting card: NOTE BUSTER_ANISO_WEIGHT SCA  16.0
 Interpreting card: NOTE BUSTER_ANISO_WEIGHT SPH   0.6    0.6
 Interpreting card: NOTE BUSTER_ANISO_WEIGHT SIM   7.8    4.8    2.6    0
 Interpreting card: NOTE BUSTER_ANISO_WEIGHT RIG  21.5   21.5    3.7    0


 After TLS and ANISO: Number of atoms with fixed/combined xyz =     0
 After TLS and ANISO: Number of atoms with fixed/combined B   =     0
 After TLS and ANISO: Number of atoms with fixed/combined OCC =  6519
 After TLS and ANISO: Number of atoms with fixed/combined BIJ =  6519
 After TLS and ANISO: Number of atoms in a TLS group          =  5553
                                     cf total number of atoms =  6519
 

 ############### Setup state vector

 No free_vect_ulim_xyz argument found: using default of  100.000
 No free_vect_llim_xyz argument found: using default of -100.000
 Number of parameters added for TLS description                 =       0
 Number of parameters to describe molecular conformation        =   26076

 ############### Setup free vector

 Number of free parameters to be adjusted in refinement   =   26076

 ############### Setting up own geometry


 No command line argument '-tnt_fn' so will use gelly to do geometry function calculations

 ============= Call TNT functions


 ============= Get TNT symmetry information

 
      Getting symmetry operators from TNT. 
          gelly will classify symmetry using pdb-like convention: 
               SYMOP   SYMMETRY
              NNNMMM   OPERATOR
                1555   X,Y,Z
                2555   -Y,X-Y,Z
                3555   -X+Y,-X,Z
                4555   -X,-Y,1/2+Z
                5555   Y,-X+Y,1/2+Z
                6555   X-Y,X,1/2+Z
          where NNN -> operator number and MMM -> translation vector
 

 ============= Own Gelly geometry/restraints setup


 ----------- Identify bond-types


    Setting up GEOMBONDREC_TYPE_HASH* to be able to map a given bond type (e.g.
    "single") to an internal integer (GBOND_TYPE=1) etc.

    Using default  GEOMBONDREC_TYPE_HASH_STR='SING* 1 DOUB* 2 TRIP* 3 AROM* 4 DELO* 4 METAL* 5':
    to override default use a NOTE BUSTER_GEOMBONDREC_TYPE_HASH_STR gelly card.

 ----------- Classify torsion into peptide omega and other


 ----------- Setup of non-bonded terms

 Ideal-distance contact term setup:
    Restraint function constants (normally these values are from NOTE BUSTER_IDEAL_CONTACT_* cards)
    Sigma for general contacts      0.200 Angs
    Sigma for 1-4 contacts          0.100 Angs (contacts across a torsion)
    Sigma for 1-5 contacts          0.150 Angs
    Sigma for 1-N contacts          0.200 Angs
    Sigma for HBOND pairs           0.100 Angs (contacts between HB_types A and D or B's)
    Contact distance increment for each atom in HBOND          -0.150 Angs 
    Contact distance increment for H-bond acceptor              0.100 Angs 
    Contact distance increment for 1-4 with an O atom          -0.050 Angs 
    Contact distance increment for 1-4 with an N atom          -0.180 Angs 
    Contact distance increment for 1-4 with an C atom          -0.370 Angs 
    Contact distance increment for 1-4 with an S atom          -0.150 Angs 
    Contact distance increment for 1-4 with an P atom          -0.150 Angs 
    Contact distance increment for 1-4 with any other atom     -0.150 Angs 
    All atoms matched for CCP4 atom type.
    Number of CCP4 atom types used is         23 (H-bound and not H-bound counted separately)

 Setting up short/ideal-distance contacts
   Using gelly special position handling. (For TNT instead rerun with argument '-special_tnt')

   No command line argument '-special_all' specified. We will auto-generate
   EXCLUDE cards only for atom-atom contacts across symmetry (i.e. on special
   position) when this involves a contact within a residue to itself.

   Have set up non-bonded exclusions - 1-3 contacts
   Atom A|27:CA         makes the most 1-3 contacts:  6

   Have set up non-bonded exclusions - 1-4 contacts
   Atom A|27:N          makes the most 1-4 contacts:  7

   Have set up non-bonded exclusions - 1-5 contacts
   Atom A|28:C.A        makes the most 1-5 contacts: 10

 ############################################################################################################
 ###################################################### setting up contact terms
 ############################################################################################################


 ============= Call Gelly versions of TNT functions

 WARNING: 1-4 contact without 1-(2,3)-4 data for A|401:C2(CIT)        and A|401:O6(CIT)        X-X
 WARNING: 1-4 contact without 1-(2,3)-4 data for A|401:O7(CIT)        and A|401:C5(CIT)        X-X
 WARNING: 1-4 contact without 1-(2,3)-4 data for A|401:O7(CIT)        and A|401:O5(CIT)        X-X
 WARNING: 1-4 contact without 1-(2,3)-4 data for A|401:C4(CIT)        and A|401:O6(CIT)        X-X
 WARNING: 1-4 contact without 1-(2,3)-4 data for A|501:C01(LIG)       and A|501:N08(LIG)       X-X
 WARNING: 1-4 contact without 1-(2,3)-4 data for A|501:C01(LIG)       and A|501:C11(LIG)       X-X
 WARNING: 1-4 contact without 1-(2,3)-4 data for A|501:N02(LIG)       and A|501:C10(LIG)       X-X
 WARNING: 1-4 contact without 1-(2,3)-4 data for B|401:C1(CIT)        and B|401:C4(CIT)        X-X
 WARNING: 1-4 contact without 1-(2,3)-4 data for B|401:C2(CIT)        and B|401:O6(CIT)        X-X
 WARNING: 1-4 contact without 1-(2,3)-4 data for B|401:O7(CIT)        and B|401:O5(CIT)        X-X
 WARNING: 1-4 contact without 1-(2,3)-4 data for B|401:C4(CIT)        and B|401:O6(CIT)        X-X

       TERM      weight      number      TNT_fn    Gelly_fn        diff
       BOND       2.000        5668    1621.868    1621.868       0.000
       ANGL       2.000        7691    5319.279    5319.279       0.000
       IMPR       0.000           0       0.000       0.000       0.000
       TORS       2.000        1973    3287.654    3287.654      -0.000
       TRIG       2.000           0       0.000       0.000       0.000
       PLAN       5.000         967    2164.073    2164.073       0.000
       CONT       5.000           0     161.209       0.000     161.209
       BCOR      10.000        5630    7703.965   15965.048   -8261.084


 ============= Call Gelly geometry functions

   Switching torsion functional form to sinusoidal rather than split harmonic functional.
      if you want this not to happen then rerun with '-torsharm'
      Weight for sinusoidal torsion=     2.000
   BCOR function/gradient will be split between H and non-H atoms (as for any atom pair). Use '-bcorrel_all_in_hydrogens' to change this.

 ----------- Fix B-correlation restraints

 
 Checking for bonds that do not have a bcorrel restraint defined
 We will wipe all existing BCORrels if OCC=0.0 atom(s) is/are involved. This can be' switched off via the '-bcorrel_nowipe_zeroocc' flag.
 We will wipe all existing BCORrels if only non-hydrogen atom(s) with fixed B(iso) are involved. This can be switched off via the '-bcorrel_nowipe_fixb_nonhydrogens' flag.
 We will keep all existing BCORrels if hydrogen atom(s) with fixed B(iso) is/are involved. This can be switched off via the '-bcorrel_wipe_fixb_hydrogens' flag.
 No -bcorrel_missing_sigma found. So will set sigma to     2.5 Ang**2 for any bond with no existing BCORrel
 No -bcorrel_missing_sigma_hydrogens found. So will set sigma to     1.0 Ang**2 for any bond with no existing BCORrel involving hydrogens

 No active BCORels found involving OCC=0.0 atom(s)


 No active BCORels found involving fixed-B (only) non-hydrogen atom(s)

    add a default BCOR for bond A|401:C1(CIT) A|401:C2(CIT)
    add a default BCOR for bond A|401:C1(CIT) A|401:O1(CIT)
    add a default BCOR for bond A|401:C1(CIT) A|401:O2(CIT)
    add a default BCOR for bond A|401:C2(CIT) A|401:C3(CIT)
    add a default BCOR for bond A|401:C3(CIT) A|401:C4(CIT)
    add a default BCOR for bond A|401:C3(CIT) A|401:C6(CIT)
    add a default BCOR for bond A|401:C3(CIT) A|401:O7(CIT)
    add a default BCOR for bond A|401:C4(CIT) A|401:C5(CIT)
    add a default BCOR for bond A|401:C5(CIT) A|401:O3(CIT)
    add a default BCOR for bond A|401:C5(CIT) A|401:O4(CIT)
    add a default BCOR for bond A|401:C6(CIT) A|401:O5(CIT)
    add a default BCOR for bond A|401:C6(CIT) A|401:O6(CIT)
    add a default BCOR for bond A|501:C01(LIG) A|501:N02(LIG)
    add a default BCOR for bond A|501:C01(LIG) A|501:C09(LIG)
    add a default BCOR for bond A|501:N02(LIG) A|501:C03(LIG)
    add a default BCOR for bond A|501:C03(LIG) A|501:C04(LIG)
    add a default BCOR for bond A|501:C03(LIG) A|501:N08(LIG)
    add a default BCOR for bond A|501:C04(LIG) A|501:C05(LIG)
    add a default BCOR for bond A|501:C05(LIG) A|501:C06(LIG)
    add a default BCOR for bond A|501:C06(LIG) A|501:C07(LIG)
    add a default BCOR for bond A|501:C07(LIG) A|501:N08(LIG)
    add a default BCOR for bond A|501:C09(LIG) A|501:C10(LIG)
    add a default BCOR for bond A|501:C09(LIG) A|501:C13(LIG)
    add a default BCOR for bond A|501:C10(LIG) A|501:C11(LIG)
    add a default BCOR for bond A|501:C11(LIG) A|501:N12(LIG)
    add a default BCOR for bond A|501:N12(LIG) A|501:C13(LIG)
    add a default BCOR for bond B|401:C1(CIT) B|401:C2(CIT)
    add a default BCOR for bond B|401:C1(CIT) B|401:O1(CIT)
    add a default BCOR for bond B|401:C1(CIT) B|401:O2(CIT)
    add a default BCOR for bond B|401:C2(CIT) B|401:C3(CIT)
    add a default BCOR for bond B|401:C3(CIT) B|401:C4(CIT)
    add a default BCOR for bond B|401:C3(CIT) B|401:C6(CIT)
    add a default BCOR for bond B|401:C3(CIT) B|401:O7(CIT)
    add a default BCOR for bond B|401:C4(CIT) B|401:C5(CIT)
    add a default BCOR for bond B|401:C5(CIT) B|401:O3(CIT)
    add a default BCOR for bond B|401:C5(CIT) B|401:O4(CIT)
    add a default BCOR for bond B|401:C6(CIT) B|401:O5(CIT)
    add a default BCOR for bond B|401:C6(CIT) B|401:O6(CIT)

 Have added a total of        38 BCORrels for bonds which do not already have a BCORrel


 No bonds (for potential BCORrel addition) were skipped due to both atoms having constant/fixed B(iso)


 ----------- Deactivate hydrogen-free planes if hydrogen atoms present

 A total of       0 planes have been deactivated because they are duplicated hydrogen-free and with-hydrogen forms.

 ----------- Handle peptide omega angle(s)

      Torsion angles split into peptide omega and other
         Number of peptide omega angles is       698
         Number of other torsion is             1275
      Peptide omega angles are monitored rather than directly restrained (PLANE used instead).

 ----------- Fix cis-PRO

 Now apply the fix to get correct restraints for cis PRO residues
   Have got      11 bonds records and      26 angles
   from GEOMETRY cards for the special PROC residue
   Weight for ideal-distance contact term=     4.000

 ----------- Prepare SSBOND/LINK for output PDB file


 ----------- Prepare for QM


 ----------- Prepare symmetry in utility distance restraints


 ############### Setting up Rhofit contacts (if needed)

 No ligand-(fixed protein) contact term will be used

 ############### Working out initial function and gradient


   MaxLik Scaling Cycle 0002
 
 Initial functional value = 3.978960E+05 rms gradient=      95.5451
 X-ray contribution to function value = 3.520348E+05
 Geometry contribution (inc ncs)      =      45861.2
 Geometry function summary
     term                           number     weight       rms        GooF      function     fn/numb
     ================================================================================================
     BOND bond lengths (angs)       5668       2.000      0.0076       0.378      1621.87       0.286
     ANGL bond angles (degs)        7691       2.000        0.99       0.588      5319.28       0.692
     TORS split-harmonic tors       1275       0.000       18.06       1.135         0.00       0.000
     SINTOR sinusoidal tors         1275       2.000       18.06                  2450.20       1.922
     PLAN general plane (angs)       967       5.000      0.0134       0.669      2164.07       2.238
     BCORrelations (angs^2)         5668      10.000       1.489       0.532     16025.60       2.827
     CONT (bad contacts)               0       5.000                                 0.00
     IDEAL(ideal-dist contact)      5530       4.000                             11921.37
     CHIRAL (gelly semiharmon)       740       5.000                                44.33
     SIM similarity restraint for NCS and/or TARGET                               6314.44
     OCCSUM restraint on sum of occupanies                                           0.00       as none defined
     DISTAN utility restraints interatomic distance                                  0.00       as none defined
     UTILANGLE utility angle restraints                                              0.00       as none defined
     UTILTOR utility restraints on torsion angles                                    0.00       as none defined
     Peptide omega torsion angles                           2.75 degs - monitor only

 Weighted rms      0.0076         0.987  (temporary rms bond and angle for BUSTER to pick up!)
 Weighted intra-residual rms(bond) for StdProtein (excluding H)      0.0074
 Weighted intra-residual rms(bond) for Buffer     (excluding H)      0.0030

 ############### Running initial check


 Initial Geometry Sanity Check

 Check for duplicate bonds: no duplicates found
 
 Check for duplicate or missing bond angles: all are OK
 Check for bond between an atom and itself not involving symmetry:  all are OK
   There are no bad contacts where Delta/sigma is greater than     6.5
   Worst contact dist=   3.066 std=   3.802 sigma=   0.206 abs(Delta/sigma)=    3.572 ats A|104:CE (MET) B|23:CB (PRO)
   It is likely that the geometry libraries, sequence info and links match the input molecule
 GEOMETRY CHECK OVERALL:  PASS


 ############### Creating geometry/screen report


 ############### Option "tao" - run Tao optimizer

 TAO: Toolkit for Advanced Optimization. Reference: 
     Munson, T., Sarich, J., Wild, S., Benson, S., McInnes, L.C. (2018)
     "TAO User Manual (Revision 3.9)", Mathematics and Computer Science
     Division, Argonne National Laboratory
     ANL/MCS-TM-322, http://www.mcs.anl.gov/petsc/
 
 Have found -keeppdb flag so will write out a .pdb file every 1000000 its during opt
 Will apply TAO optimization 
 Maximum number of iterations      100
 Convergence test on Grms       4.0000 (-glim)
 Limit on rms displacement to original position (rmsD2init): NONE test inactive (-dlim)

    analysing argument no 0 : -autoncs with j=0
    analysing argument no 1 : -hydrogens_input_occ2zero with j=0
    analysing argument no 2 : -hydrogens_output_occ2heavy with j=0
    analysing argument no 3 : -screen with j=0
    analysing argument no 4 : 100 with j=0
    analysing argument no 5 : -screen_sigma with j=0
    analysing argument no 6 : 3.0 with j=0
    analysing argument no 7 : -keeppdb with j=0
    analysing argument no 8 : 1000000 with j=0
    analysing argument no 9 : -taomonitor_verbose with j=0
    found argument starting with "-tao" : -taomonitor_verbose
    analysing argument no 10 : 2 with j=1
    found value after -tao/-options_/-viewer_ argument : "2"
 PETSC/TAO: setting option "-taomonitor_verbose" = "2"


   MaxLik Scaling Cycle 0003
   Iters     Tot_Funct    Xray_Funct    Geom_Funct          Grms     rmsD2init     maxD2init             R         Rfree           LLG       LLGfree     rms(bond)    rms(angle)       Opttag_H
   Iters             K   B_IMPF_FRAG        K_MISS   B_TEMP_MISS   K_IMPF_MISS   B_IMPF_MISS        K_SOLV   B_TEMP_SOLV   K_IMPF_SOLV   B_IMPF_SOLV        B_TEMP     B_TEMP_11     B_TEMP_12     B_TEMP_22     B_TEMP_13     B_TEMP_23     B_TEMP_33      K_WILSON      B_WILSON      FGCYCLES       Scatag_H
   Iters          BOND         ANGLE      IMPROPER       TORSION    SINTORSION      TRIGONAL        PLANAR  BCORRELATION       CONTACT     IDEALDIST        CHIRAL    SIMILARITY       OCCSSUM  UTILDISTANCE     UTILANGLE   UTILTORSION        RHOFIT        ADP-01        ADP-02        ADP-03        ADP-04        ADP-05        ADP-06        ADP-07        ADP-08        ADP-09        ADP-10      RHOFITPC            QM       Funtag_H
       0  3.978388E+05  3.520328E+05       45806.0       92.5080  0.000000E+00  0.000000E+00      0.188930      0.242767  0.000000E+00  0.000000E+00    0.00758397      0.986951       Opttag_D
       0       81.9577       1.35437       1.00000  0.000000E+00       1.00000  0.000000E+00      0.947011       104.199       1.00000       37.1410  0.000000E+00      0.682793  0.000000E+00      0.682793  0.000000E+00  0.000000E+00      -1.36559       82.1558      0.672497        2       Scatag_D
       0       1621.87       5319.28  0.000000E+00  0.000000E+00       2450.20  0.000000E+00       2164.07       15970.4  0.000000E+00       11921.4       44.3271       6314.44 -1.000000E+10 -1.000000E+10 -1.000000E+10 -1.000000E+10 -1.000000E+10  0.000000E+00  0.000000E+00  0.000000E+00  0.000000E+00  0.000000E+00  0.000000E+00  0.000000E+00  0.000000E+00  0.000000E+00  0.000000E+00  0.000000E+00  0.000000E+00       Funtag_D
   Geometry Restraint Deviations SCREEN Information

 

   MaxLik Scaling Cycle 0004

   MaxLik Scaling Cycle 0005

   Wilson plot

   Statistics in resolution bins after refinement cycle 0001
```


|  |  |
| --- | --- |
|  | - Rxpct factors plot- Rxpct factors table- <Log-Lik Gain>- Ln Sigma A- Correlation Coefficients |


```
   Iters     Tot_Funct    Xray_Funct    Geom_Funct          Grms     rmsD2init     maxD2init             R         Rfree           LLG       LLGfree     rms(bond)    rms(angle)
   Iters             K   B_IMPF_FRAG        K_MISS   B_TEMP_MISS   K_IMPF_MISS   B_IMPF_MISS        K_SOLV   B_TEMP_SOLV   K_IMPF_SOLV   B_IMPF_SOLV        B_TEMP     B_TEMP_11     B_TEMP_12     B_TEMP_22     B_TEMP_13     B_TEMP_23     B_TEMP_33      K_WILSON      B_WILSON      FGCYCLES
   Iters          BOND         ANGLE      IMPROPER       TORSION    SINTORSION      TRIGONAL        PLANAR  BCORRELATION       CONTACT     IDEALDIST        CHIRAL    SIMILARITY       OCCSSUM  UTILDISTANCE     UTILANGLE   UTILTORSION        RHOFIT        ADP-01        ADP-02        ADP-03        ADP-04        ADP-05        ADP-06        ADP-07        ADP-08        ADP-09        ADP-10      RHOFITPC            QM
       1  3.973951E+05  3.517836E+05       45611.5       150.924  8.840521E-04    0.00689103      0.188834      0.242807  6.845135E-04 -8.495417E-04    0.00759274      0.988204       Opttag_D
       1       81.8918       1.34021       1.00000  0.000000E+00       1.00000  0.000000E+00      0.947071       103.869       1.00000       37.0822  0.000000E+00      0.691590  0.000000E+00      0.691590  0.000000E+00  0.000000E+00      -1.38318       82.1558      0.672497        2       Scatag_D
       1       1625.62       5332.80  0.000000E+00  0.000000E+00       2450.25  0.000000E+00       2161.58       15763.2  0.000000E+00       11926.7       44.6128       6306.79 -1.000000E+10 -1.000000E+10 -1.000000E+10 -1.000000E+10 -1.000000E+10  0.000000E+00  0.000000E+00  0.000000E+00  0.000000E+00  0.000000E+00  0.000000E+00  0.000000E+00  0.000000E+00  0.000000E+00  0.000000E+00  0.000000E+00  0.000000E+00       Funtag_D
   Geometry Restraint Deviations SCREEN Information

 

   MaxLik Scaling Cycle 0006

   Wilson plot

   Statistics in resolution bins after refinement cycle 0002
```


|  |  |
| --- | --- |
|  | - Rxpct factors plot- Rxpct factors table- <Log-Lik Gain>- Ln Sigma A- Correlation Coefficients |


```
   Iters     Tot_Funct    Xray_Funct    Geom_Funct          Grms     rmsD2init     maxD2init             R         Rfree           LLG       LLGfree     rms(bond)    rms(angle)
   Iters             K   B_IMPF_FRAG        K_MISS   B_TEMP_MISS   K_IMPF_MISS   B_IMPF_MISS        K_SOLV   B_TEMP_SOLV   K_IMPF_SOLV   B_IMPF_SOLV        B_TEMP     B_TEMP_11     B_TEMP_12     B_TEMP_22     B_TEMP_13     B_TEMP_23     B_TEMP_33      K_WILSON      B_WILSON      FGCYCLES
   Iters          BOND         ANGLE      IMPROPER       TORSION    SINTORSION      TRIGONAL        PLANAR  BCORRELATION       CONTACT     IDEALDIST        CHIRAL    SIMILARITY       OCCSSUM  UTILDISTANCE     UTILANGLE   UTILTORSION        RHOFIT        ADP-01        ADP-02        ADP-03        ADP-04        ADP-05        ADP-06        ADP-07        ADP-08        ADP-09        ADP-10      RHOFITPC            QM
       2  3.951701E+05  3.517546E+05       43415.5       51.8700  9.566369E-04    0.00682768      0.188817      0.242797  7.680578E-04 -9.444451E-04    0.00758623      0.987554       Opttag_D
       2       81.8816       1.33843       1.00000  0.000000E+00       1.00000  0.000000E+00      0.947027       103.859       1.00000       37.0687  0.000000E+00      0.693284  0.000000E+00      0.693284  0.000000E+00  0.000000E+00      -1.38657       82.1558      0.672497        1       Scatag_D
       2       1622.84       5325.78  0.000000E+00  0.000000E+00       2450.24  0.000000E+00       2161.35       13576.9  0.000000E+00       11927.4       44.5080       6306.45 -1.000000E+10 -1.000000E+10 -1.000000E+10 -1.000000E+10 -1.000000E+10  0.000000E+00  0.000000E+00  0.000000E+00  0.000000E+00  0.000000E+00  0.000000E+00  0.000000E+00  0.000000E+00  0.000000E+00  0.000000E+00  0.000000E+00  0.000000E+00       Funtag_D
   Geometry Restraint Deviations SCREEN Information

 
       3  3.947742E+05  3.516853E+05       43088.9       41.8807    0.00120458    0.00786613      0.188785      0.242787  9.683809E-04   -0.00104237    0.00758981      0.988331       Opttag_D
       3       81.8594       1.33428       1.00000  0.000000E+00       1.00000  0.000000E+00      0.946989       103.792       1.00000       37.0442  0.000000E+00      0.696633  0.000000E+00      0.696633  0.000000E+00  0.000000E+00      -1.39327       82.1558      0.672497        1       Scatag_D
       3       1624.37       5334.17  0.000000E+00  0.000000E+00       2450.34  0.000000E+00       2160.85       13239.9  0.000000E+00       11929.8       44.4858       6305.06 -1.000000E+10 -1.000000E+10 -1.000000E+10 -1.000000E+10 -1.000000E+10  0.000000E+00  0.000000E+00  0.000000E+00  0.000000E+00  0.000000E+00  0.000000E+00  0.000000E+00  0.000000E+00  0.000000E+00  0.000000E+00  0.000000E+00  0.000000E+00       Funtag_D
       4  3.942466E+05  3.515180E+05       42728.6       41.1038    0.00182665     0.0112358      0.188706      0.242773    0.00145125   -0.00131355    0.00760652      0.987105       Opttag_D
       4       81.8037       1.32414       1.00000  0.000000E+00       1.00000  0.000000E+00      0.946913       103.609       1.00000       36.9851  0.000000E+00      0.705473  0.000000E+00      0.705473  0.000000E+00  0.000000E+00      -1.41095       82.1558      0.672497        1       Scatag_D
       4       1631.53       5320.94  0.000000E+00  0.000000E+00       2450.56  0.000000E+00       2159.91       12879.6  0.000000E+00       11935.6       44.4858       6305.96 -1.000000E+10 -1.000000E+10 -1.000000E+10 -1.000000E+10 -1.000000E+10  0.000000E+00  0.000000E+00  0.000000E+00  0.000000E+00  0.000000E+00  0.000000E+00  0.000000E+00  0.000000E+00  0.000000E+00  0.000000E+00  0.000000E+00  0.000000E+00       Funtag_D
       5  3.936914E+05  3.512185E+05       42472.8       60.3882    0.00312100     0.0190222      0.188574      0.242759    0.00231617   -0.00182333    0.00769675      0.995021       Opttag_D
       5       81.6929       1.30530       1.00000  0.000000E+00       1.00000  0.000000E+00      0.946775       103.235       1.00000       36.8685  0.000000E+00      0.724766  0.000000E+00      0.724766  0.000000E+00  0.000000E+00      -1.44953       82.1558      0.672497        1       Scatag_D
       5       1670.46       5406.62  0.000000E+00  0.000000E+00       2451.07  0.000000E+00       2159.51       12473.8  0.000000E+00       11950.5       44.5067       6316.36 -1.000000E+10 -1.000000E+10 -1.000000E+10 -1.000000E+10 -1.000000E+10  0.000000E+00  0.000000E+00  0.000000E+00  0.000000E+00  0.000000E+00  0.000000E+00  0.000000E+00  0.000000E+00  0.000000E+00  0.000000E+00  0.000000E+00  0.000000E+00       Funtag_D
       6  3.932149E+05  3.510227E+05       42192.2       49.7480    0.00407328     0.0257459      0.188497      0.242764    0.00288166   -0.00222890    0.00773428      0.993773       Opttag_D
       6       81.6071       1.29213       1.00000  0.000000E+00       1.00000  0.000000E+00      0.946643       102.966       1.00000       36.7788  0.000000E+00      0.740643  0.000000E+00      0.740643  0.000000E+00  0.000000E+00      -1.48129       82.1558      0.672497        1       Scatag_D
       6       1686.80       5393.07  0.000000E+00  0.000000E+00       2451.60  0.000000E+00       2160.44       12167.8  0.000000E+00       11960.8       44.4983       6327.16 -1.000000E+10 -1.000000E+10 -1.000000E+10 -1.000000E+10 -1.000000E+10  0.000000E+00  0.000000E+00  0.000000E+00  0.000000E+00  0.000000E+00  0.000000E+00  0.000000E+00  0.000000E+00  0.000000E+00  0.000000E+00  0.000000E+00  0.000000E+00       Funtag_D
       7  3.928220E+05  3.509066E+05       41915.4       37.1078    0.00467368     0.0307532      0.188456      0.242768    0.00321696   -0.00249441    0.00774486      0.990920       Opttag_D
       7       81.5499       1.28400       1.00000  0.000000E+00       1.00000  0.000000E+00      0.946542       102.800       1.00000       36.7179  0.000000E+00      0.752109  0.000000E+00      0.752109  0.000000E+00  0.000000E+00      -1.50422       82.1558      0.672497        1       Scatag_D
       7       1691.41       5362.14  0.000000E+00  0.000000E+00       2451.99  0.000000E+00       2161.50       11904.6  0.000000E+00       11967.4       44.4690       6331.84 -1.000000E+10 -1.000000E+10 -1.000000E+10 -1.000000E+10 -1.000000E+10  0.000000E+00  0.000000E+00  0.000000E+00  0.000000E+00  0.000000E+00  0.000000E+00  0.000000E+00  0.000000E+00  0.000000E+00  0.000000E+00  0.000000E+00  0.000000E+00       Funtag_D
       8  3.923659E+05  3.507465E+05       41619.4       40.2446    0.00566627     0.0397553      0.188410      0.242794    0.00367930   -0.00290011    0.00775424      0.991952       Opttag_D
       8       81.4565       1.27188       1.00000  0.000000E+00       1.00000  0.000000E+00      0.946356       102.547       1.00000       36.6183  0.000000E+00      0.771444  0.000000E+00      0.771444  0.000000E+00  0.000000E+00      -1.54289       82.1558      0.672497        1       Scatag_D
       8       1695.51       5373.32  0.000000E+00  0.000000E+00       2452.68  0.000000E+00       2164.12       11575.0  0.000000E+00       11977.1       44.4790       6337.21 -1.000000E+10 -1.000000E+10 -1.000000E+10 -1.000000E+10 -1.000000E+10  0.000000E+00  0.000000E+00  0.000000E+00  0.000000E+00  0.000000E+00  0.000000E+00  0.000000E+00  0.000000E+00  0.000000E+00  0.000000E+00  0.000000E+00  0.000000E+00       Funtag_D
       9  3.921338E+05  3.505584E+05       41575.4       79.6716    0.00750134     0.0568336      0.188385      0.242892    0.00422254   -0.00348969    0.00784261       1.00981       Opttag_D
       9       81.2988       1.25414       1.00000  0.000000E+00       1.00000  0.000000E+00      0.946029       102.121       1.00000       36.4588  0.000000E+00      0.803692  0.000000E+00      0.803692  0.000000E+00  0.000000E+00      -1.60738       82.1558      0.672497        1       Scatag_D
       9       1734.38       5568.49  0.000000E+00  0.000000E+00       2453.91  0.000000E+00       2171.16       11253.9  0.000000E+00       11995.8       44.7163       6353.10 -1.000000E+10 -1.000000E+10 -1.000000E+10 -1.000000E+10 -1.000000E+10  0.000000E+00  0.000000E+00  0.000000E+00  0.000000E+00  0.000000E+00  0.000000E+00  0.000000E+00  0.000000E+00  0.000000E+00  0.000000E+00  0.000000E+00  0.000000E+00       Funtag_D
      10  3.916939E+05  3.505419E+05       41152.0       34.4167    0.00768075     0.0596190      0.188386      0.242908    0.00427010   -0.00356346    0.00776823      0.992306       Opttag_D
      10       81.2793       1.25232       1.00000  0.000000E+00       1.00000  0.000000E+00      0.945979       102.080       1.00000       36.4377  0.000000E+00      0.807861  0.000000E+00      0.807861  0.000000E+00  0.000000E+00      -1.61572       82.1558      0.672497        1       Scatag_D
      10       1701.64       5377.15  0.000000E+00  0.000000E+00       2454.03  0.000000E+00       2172.21       11057.7  0.000000E+00       11996.6       44.7581       6347.91 -1.000000E+10 -1.000000E+10 -1.000000E+10 -1.000000E+10 -1.000000E+10  0.000000E+00  0.000000E+00  0.000000E+00  0.000000E+00  0.000000E+00  0.000000E+00  0.000000E+00  0.000000E+00  0.000000E+00  0.000000E+00  0.000000E+00  0.000000E+00       Funtag_D
      11  3.914712E+05  3.505172E+05       40954.0       28.0648    0.00800425     0.0641316      0.188388      0.242932    0.00434145   -0.00366090    0.00775197      0.991910       Opttag_D
      11       81.2490       1.24944       1.00000  0.000000E+00       1.00000  0.000000E+00      0.945907       102.011       1.00000       36.4056  0.000000E+00      0.814014  0.000000E+00      0.814014  0.000000E+00  0.000000E+00      -1.62803       82.1558      0.672497        1       Scatag_D
      11       1694.52       5372.87  0.000000E+00  0.000000E+00       2454.19  0.000000E+00       2173.94       10871.1  0.000000E+00       11998.0       44.8361       6344.49 -1.000000E+10 -1.000000E+10 -1.000000E+10 -1.000000E+10 -1.000000E+10  0.000000E+00  0.000000E+00  0.000000E+00  0.000000E+00  0.000000E+00  0.000000E+00  0.000000E+00  0.000000E+00  0.000000E+00  0.000000E+00  0.000000E+00  0.000000E+00       Funtag_D
      12  3.912246E+05  3.504682E+05       40756.4       35.2918    0.00880088     0.0740829      0.188397      0.242993    0.00448283   -0.00388041    0.00774656      0.993607       Opttag_D
      12       81.1813       1.24307       1.00000  0.000000E+00       1.00000  0.000000E+00      0.945760       101.837       1.00000       36.3359  0.000000E+00      0.827663  0.000000E+00      0.827663  0.000000E+00  0.000000E+00      -1.65533       82.1558      0.672497        1       Scatag_D
      12       1692.15       5391.27  0.000000E+00  0.000000E+00       2454.56  0.000000E+00       2177.98       10649.0  0.000000E+00       12003.4       44.9923       6342.97 -1.000000E+10 -1.000000E+10 -1.000000E+10 -1.000000E+10 -1.000000E+10  0.000000E+00  0.000000E+00  0.000000E+00  0.000000E+00  0.000000E+00  0.000000E+00  0.000000E+00  0.000000E+00  0.000000E+00  0.000000E+00  0.000000E+00  0.000000E+00       Funtag_D
      13  3.909927E+05  3.503991E+05       40593.6       65.1451     0.0104679     0.0954510      0.188433      0.243118    0.00468244   -0.00420533    0.00778832       1.00512       Opttag_D
      13       81.0521       1.23151       1.00000  0.000000E+00       1.00000  0.000000E+00      0.945493       101.482       1.00000       36.2030  0.000000E+00      0.853264  0.000000E+00      0.853264  0.000000E+00  0.000000E+00      -1.70653       82.1558      0.672497        1       Scatag_D
      13       1710.45       5516.98  0.000000E+00  0.000000E+00       2455.08  0.000000E+00       2186.79       10319.5  0.000000E+00       12016.1       45.3039       6343.41 -1.000000E+10 -1.000000E+10 -1.000000E+10 -1.000000E+10 -1.000000E+10  0.000000E+00  0.000000E+00  0.000000E+00  0.000000E+00  0.000000E+00  0.000000E+00  0.000000E+00  0.000000E+00  0.000000E+00  0.000000E+00  0.000000E+00  0.000000E+00       Funtag_D
      14  3.906760E+05  3.503801E+05       40295.9       30.2539     0.0112563      0.105538      0.188456      0.243189    0.00473752   -0.00434503    0.00775768      0.993204       Opttag_D
      14       80.9948       1.22661       1.00000  0.000000E+00       1.00000  0.000000E+00      0.945374       101.316       1.00000       36.1446  0.000000E+00      0.864165  0.000000E+00      0.864165  0.000000E+00  0.000000E+00      -1.72833       82.1558      0.672497        1       Scatag_D
      14       1697.02       5386.89  0.000000E+00  0.000000E+00       2455.29  0.000000E+00       2190.71       10155.0  0.000000E+00       12022.4       45.4270       6343.17 -1.000000E+10 -1.000000E+10 -1.000000E+10 -1.000000E+10 -1.000000E+10  0.000000E+00  0.000000E+00  0.000000E+00  0.000000E+00  0.000000E+00  0.000000E+00  0.000000E+00  0.000000E+00  0.000000E+00  0.000000E+00  0.000000E+00  0.000000E+00       Funtag_D
      15  3.904933E+05  3.503510E+05       40142.2       23.0644     0.0118111      0.113615      0.188463      0.243209    0.00482127   -0.00438427    0.00776354      0.992977       Opttag_D
      15       80.9556       1.22270       1.00000  0.000000E+00       1.00000  0.000000E+00      0.945299       101.193       1.00000       36.1002  0.000000E+00      0.870561  0.000000E+00      0.870561  0.000000E+00  0.000000E+00      -1.74112       82.1558      0.672497        1       Scatag_D
      15       1699.58       5384.43  0.000000E+00  0.000000E+00       2455.29  0.000000E+00       2192.67       9995.79  0.000000E+00       12026.6       45.4216       6342.50 -1.000000E+10 -1.000000E+10 -1.000000E+10 -1.000000E+10 -1.000000E+10  0.000000E+00  0.000000E+00  0.000000E+00  0.000000E+00  0.000000E+00  0.000000E+00  0.000000E+00  0.000000E+00  0.000000E+00  0.000000E+00  0.000000E+00  0.000000E+00       Funtag_D
      16  3.903114E+05  3.503061E+05       40005.3       28.0971     0.0127167      0.127130      0.188480      0.243227    0.00495116   -0.00441952    0.00777628      0.993991       Opttag_D
      16       80.8955       1.21656       1.00000  0.000000E+00       1.00000  0.000000E+00      0.945179       101.000       1.00000       36.0306  0.000000E+00      0.879791  0.000000E+00      0.879791  0.000000E+00  0.000000E+00      -1.75958       82.1558      0.672497        1       Scatag_D
      16       1705.17       5395.44  0.000000E+00  0.000000E+00       2455.20  0.000000E+00       2195.50       9832.84  0.000000E+00       12033.9       45.4213       6341.80 -1.000000E+10 -1.000000E+10 -1.000000E+10 -1.000000E+10 -1.000000E+10  0.000000E+00  0.000000E+00  0.000000E+00  0.000000E+00  0.000000E+00  0.000000E+00  0.000000E+00  0.000000E+00  0.000000E+00  0.000000E+00  0.000000E+00  0.000000E+00       Funtag_D
      17  3.901821E+05  3.502454E+05       39936.7       54.7460     0.0143648      0.151986      0.188518      0.243266    0.00512628   -0.00449668    0.00782779       1.00231       Opttag_D
      17       80.7964       1.20657       1.00000  0.000000E+00       1.00000  0.000000E+00      0.944970       100.665       1.00000       35.9168  0.000000E+00      0.893849  0.000000E+00      0.893849  0.000000E+00  0.000000E+00      -1.78770       82.1558      0.672497        1       Scatag_D
      17       1727.83       5486.14  0.000000E+00  0.000000E+00       2455.22  0.000000E+00       2199.88       9631.45  0.000000E+00       12047.8       45.4965       6342.84 -1.000000E+10 -1.000000E+10 -1.000000E+10 -1.000000E+10 -1.000000E+10  0.000000E+00  0.000000E+00  0.000000E+00  0.000000E+00  0.000000E+00  0.000000E+00  0.000000E+00  0.000000E+00  0.000000E+00  0.000000E+00  0.000000E+00  0.000000E+00       Funtag_D
      18  3.899795E+05  3.502235E+05       39756.0       26.0628     0.0147742      0.158892      0.188521      0.243268    0.00518960   -0.00451346    0.00780361      0.993502       Opttag_D
      18       80.7743       1.20396       1.00000  0.000000E+00       1.00000  0.000000E+00      0.944923       100.582       1.00000       35.8890  0.000000E+00      0.896154  0.000000E+00      0.896154  0.000000E+00  0.000000E+00      -1.79231       82.1558      0.672497        1       Scatag_D
      18       1717.17       5390.12  0.000000E+00  0.000000E+00       2455.03  0.000000E+00       2200.02       9555.03  0.000000E+00       12051.5       45.5188       6341.63 -1.000000E+10 -1.000000E+10 -1.000000E+10 -1.000000E+10 -1.000000E+10  0.000000E+00  0.000000E+00  0.000000E+00  0.000000E+00  0.000000E+00  0.000000E+00  0.000000E+00  0.000000E+00  0.000000E+00  0.000000E+00  0.000000E+00  0.000000E+00       Funtag_D
      19  3.898400E+05  3.501893E+05       39650.7       20.6321     0.0153232      0.168520      0.188526      0.243273    0.00528818   -0.00457420    0.00780478      0.993491       Opttag_D
      19       80.7472       1.20042       1.00000  0.000000E+00       1.00000  0.000000E+00      0.944861       100.473       1.00000       35.8531  0.000000E+00      0.898398  0.000000E+00      0.898398  0.000000E+00  0.000000E+00      -1.79680       82.1558      0.672497        1       Scatag_D
      19       1717.69       5390.00  0.000000E+00  0.000000E+00       2454.90  0.000000E+00       2199.36       9446.16  0.000000E+00       12056.4       45.5711       6340.61 -1.000000E+10 -1.000000E+10 -1.000000E+10 -1.000000E+10 -1.000000E+10  0.000000E+00  0.000000E+00  0.000000E+00  0.000000E+00  0.000000E+00  0.000000E+00  0.000000E+00  0.000000E+00  0.000000E+00  0.000000E+00  0.000000E+00  0.000000E+00       Funtag_D
      20  3.897178E+05  3.501505E+05       39567.2       22.9342     0.0161634      0.182966      0.188538      0.243298    0.00540029   -0.00468950    0.00781497      0.994145       Opttag_D
      20       80.7085       1.19559       1.00000  0.000000E+00       1.00000  0.000000E+00      0.944774       100.312       1.00000       35.8031  0.000000E+00      0.901788  0.000000E+00      0.901788  0.000000E+00  0.000000E+00      -1.80358       82.1558      0.672497        1       Scatag_D
      20       1722.17       5397.11  0.000000E+00  0.000000E+00       2454.82  0.000000E+00       2198.74       9344.99  0.000000E+00       12063.5       45.6678       6340.21 -1.000000E+10 -1.000000E+10 -1.000000E+10 -1.000000E+10 -1.000000E+10  0.000000E+00  0.000000E+00  0.000000E+00  0.000000E+00  0.000000E+00  0.000000E+00  0.000000E+00  0.000000E+00  0.000000E+00  0.000000E+00  0.000000E+00  0.000000E+00       Funtag_D
      21  3.896338E+05  3.500803E+05       39553.5       52.9537     0.0178646      0.212896      0.188561      0.243358    0.00560315   -0.00495272    0.00785532       1.00385       Opttag_D
      21       80.6433       1.18676       1.00000  0.000000E+00       1.00000  0.000000E+00      0.944628       99.9944       1.00000       35.7128  0.000000E+00      0.906355  0.000000E+00      0.906355  0.000000E+00  0.000000E+00      -1.81271       82.1558      0.672497        1       Scatag_D
      21       1740.00       5502.98  0.000000E+00  0.000000E+00       2454.96  0.000000E+00       2196.52       9195.01  0.000000E+00       12077.9       45.8612       6340.30 -1.000000E+10 -1.000000E+10 -1.000000E+10 -1.000000E+10 -1.000000E+10  0.000000E+00  0.000000E+00  0.000000E+00  0.000000E+00  0.000000E+00  0.000000E+00  0.000000E+00  0.000000E+00  0.000000E+00  0.000000E+00  0.000000E+00  0.000000E+00       Funtag_D
      22  3.894621E+05  3.500644E+05       39397.7       21.6014     0.0183771      0.222065      0.188572      0.243379    0.00564909   -0.00503954    0.00783144      0.994436       Opttag_D
      22       80.6261       1.18439       1.00000  0.000000E+00       1.00000  0.000000E+00      0.944586       99.9029       1.00000       35.6889  0.000000E+00      0.907430  0.000000E+00      0.907430  0.000000E+00  0.000000E+00      -1.81486       82.1558      0.672497        1       Scatag_D
      22       1729.44       5400.26  0.000000E+00  0.000000E+00       2455.02  0.000000E+00       2196.02       9149.85  0.000000E+00       12081.1       45.9202       6340.07 -1.000000E+10 -1.000000E+10 -1.000000E+10 -1.000000E+10 -1.000000E+10  0.000000E+00  0.000000E+00  0.000000E+00  0.000000E+00  0.000000E+00  0.000000E+00  0.000000E+00  0.000000E+00  0.000000E+00  0.000000E+00  0.000000E+00  0.000000E+00       Funtag_D
      23  3.893757E+05  3.500418E+05       39333.9       15.6600     0.0187154      0.228965      0.188568      0.243386    0.00571434   -0.00511089    0.00782787      0.994043       Opttag_D
      23       80.6187       1.18263       1.00000  0.000000E+00       1.00000  0.000000E+00      0.944567       99.8435       1.00000       35.6735  0.000000E+00      0.907165  0.000000E+00      0.907165  0.000000E+00  0.000000E+00      -1.81433       82.1558      0.672497        1       Scatag_D
      23       1727.87       5396.00  0.000000E+00  0.000000E+00       2455.17  0.000000E+00       2194.79       9092.75  0.000000E+00       12082.4       45.9336       6338.96 -1.000000E+10 -1.000000E+10 -1.000000E+10 -1.000000E+10 -1.000000E+10  0.000000E+00  0.000000E+00  0.000000E+00  0.000000E+00  0.000000E+00  0.000000E+00  0.000000E+00  0.000000E+00  0.000000E+00  0.000000E+00  0.000000E+00  0.000000E+00       Funtag_D
      24  3.892698E+05  3.499927E+05       39277.1       21.5209     0.0195995      0.246152      0.188567      0.243393    0.00585602   -0.00528199    0.00782691      0.995307       Opttag_D
      24       80.6000       1.17841       1.00000  0.000000E+00       1.00000  0.000000E+00      0.944516       99.6950       1.00000       35.6354  0.000000E+00      0.906725  0.000000E+00      0.906725  0.000000E+00  0.000000E+00      -1.81345       82.1558      0.672497        1       Scatag_D
      24       1727.44       5409.73  0.000000E+00  0.000000E+00       2455.49  0.000000E+00       2192.76       9020.08  0.000000E+00       12087.2       45.9777       6338.41 -1.000000E+10 -1.000000E+10 -1.000000E+10 -1.000000E+10 -1.000000E+10  0.000000E+00  0.000000E+00  0.000000E+00  0.000000E+00  0.000000E+00  0.000000E+00  0.000000E+00  0.000000E+00  0.000000E+00  0.000000E+00  0.000000E+00  0.000000E+00       Funtag_D
      25  3.891850E+05  3.499124E+05       39272.6       40.9263     0.0211693      0.276238      0.188568      0.243393    0.00608795   -0.00560425    0.00783428       1.00084       Opttag_D
      25       80.5750       1.17159       1.00000  0.000000E+00       1.00000  0.000000E+00      0.944433       99.4481       1.00000       35.5765  0.000000E+00      0.904824  0.000000E+00      0.904824  0.000000E+00  0.000000E+00      -1.80965       82.1558      0.672497        1       Scatag_D
      25       1730.69       5470.09  0.000000E+00  0.000000E+00       2456.17  0.000000E+00       2190.75       8943.87  0.000000E+00       12096.4       46.0258       6338.61 -1.000000E+10 -1.000000E+10 -1.000000E+10 -1.000000E+10 -1.000000E+10  0.000000E+00  0.000000E+00  0.000000E+00  0.000000E+00  0.000000E+00  0.000000E+00  0.000000E+00  0.000000E+00  0.000000E+00  0.000000E+00  0.000000E+00  0.000000E+00       Funtag_D
      26  3.890648E+05  3.498786E+05       39186.2       20.0055     0.0218910      0.289867      0.188569      0.243391    0.00618542   -0.00576677    0.00782080      0.996009       Opttag_D
      26       80.5661       1.16868       1.00000  0.000000E+00       1.00000  0.000000E+00      0.944396       99.3413       1.00000       35.5524  0.000000E+00      0.903657  0.000000E+00      0.903657  0.000000E+00  0.000000E+00      -1.80731       82.1558      0.672497        1       Scatag_D
      26       1724.75       5417.37  0.000000E+00  0.000000E+00       2456.43  0.000000E+00       2190.50       8911.14  0.000000E+00       12100.4       46.0406       6339.57 -1.000000E+10 -1.000000E+10 -1.000000E+10 -1.000000E+10 -1.000000E+10  0.000000E+00  0.000000E+00  0.000000E+00  0.000000E+00  0.000000E+00  0.000000E+00  0.000000E+00  0.000000E+00  0.000000E+00  0.000000E+00  0.000000E+00  0.000000E+00       Funtag_D
      27  3.889740E+05  3.498360E+05       39138.0       15.1028     0.0226736      0.304886      0.188567      0.243377    0.00630870   -0.00597855    0.00781904      0.995964       Opttag_D
      27       80.5607       1.16555       1.00000  0.000000E+00       1.00000  0.000000E+00      0.944358       99.2326       1.00000       35.5285  0.000000E+00      0.901599  0.000000E+00      0.901599  0.000000E+00  0.000000E+00      -1.80320       82.1558      0.672497        1       Scatag_D
      27       1723.97       5416.87  0.000000E+00  0.000000E+00       2456.78  0.000000E+00       2190.75       8858.43  0.000000E+00       12104.4       46.0402       6340.74 -1.000000E+10 -1.000000E+10 -1.000000E+10 -1.000000E+10 -1.000000E+10  0.000000E+00  0.000000E+00  0.000000E+00  0.000000E+00  0.000000E+00  0.000000E+00  0.000000E+00  0.000000E+00  0.000000E+00  0.000000E+00  0.000000E+00  0.000000E+00       Funtag_D
      28  3.888835E+05  3.497773E+05       39106.2       18.6192     0.0238135      0.326640      0.188564      0.243359    0.00647803   -0.00629586    0.00782406      0.996966       Opttag_D
      28       80.5572       1.16131       1.00000  0.000000E+00       1.00000  0.000000E+00      0.944309       99.0824       1.00000       35.4980  0.000000E+00      0.897928  0.000000E+00      0.897928  0.000000E+00  0.000000E+00      -1.79586       82.1558      0.672497        1       Scatag_D
      28       1726.18       5427.78  0.000000E+00  0.000000E+00       2457.32  0.000000E+00       2191.63       8804.54  0.000000E+00       12110.0       46.0389       6342.68 -1.000000E+10 -1.000000E+10 -1.000000E+10 -1.000000E+10 -1.000000E+10  0.000000E+00  0.000000E+00  0.000000E+00  0.000000E+00  0.000000E+00  0.000000E+00  0.000000E+00  0.000000E+00  0.000000E+00  0.000000E+00  0.000000E+00  0.000000E+00       Funtag_D
      29  3.888158E+05  3.496784E+05       39137.4       41.6786     0.0260406      0.368439      0.188564      0.243315    0.00676373   -0.00687614    0.00784674       1.00369       Opttag_D
      29       80.5622       1.15414       1.00000  0.000000E+00       1.00000  0.000000E+00      0.944221       98.8155       1.00000       35.4510  0.000000E+00      0.889439  0.000000E+00      0.889439  0.000000E+00  0.000000E+00      -1.77888       82.1558      0.672497        1       Scatag_D
      29       1736.20       5501.29  0.000000E+00  0.000000E+00       2458.48  0.000000E+00       2194.90       8731.21  0.000000E+00       12121.0       46.0289       6348.29 -1.000000E+10 -1.000000E+10 -1.000000E+10 -1.000000E+10 -1.000000E+10  0.000000E+00  0.000000E+00  0.000000E+00  0.000000E+00  0.000000E+00  0.000000E+00  0.000000E+00  0.000000E+00  0.000000E+00  0.000000E+00  0.000000E+00  0.000000E+00       Funtag_D
      30  3.887001E+05  3.496490E+05       39051.1       16.5713     0.0267583      0.381881      0.188566      0.243293    0.00684869   -0.00705400    0.00783764      0.997739       Opttag_D
      30       80.5678       1.15215       1.00000  0.000000E+00       1.00000  0.000000E+00      0.944198       98.7389       1.00000       35.4386  0.000000E+00      0.886307  0.000000E+00      0.886307  0.000000E+00  0.000000E+00      -1.77261       82.1558      0.672497        1       Scatag_D
      30       1732.18       5436.20  0.000000E+00  0.000000E+00       2458.87  0.000000E+00       2196.24       8708.02  0.000000E+00       12124.5       46.0020       6349.11 -1.000000E+10 -1.000000E+10 -1.000000E+10 -1.000000E+10 -1.000000E+10  0.000000E+00  0.000000E+00  0.000000E+00  0.000000E+00  0.000000E+00  0.000000E+00  0.000000E+00  0.000000E+00  0.000000E+00  0.000000E+00  0.000000E+00  0.000000E+00       Funtag_D
      31  3.886373E+05  3.496242E+05       39013.1       12.8363     0.0271768      0.390150      0.188561      0.243263    0.00692004   -0.00714347    0.00784057      0.997374       Opttag_D
      31       80.5773       1.15112       1.00000  0.000000E+00       1.00000  0.000000E+00      0.944187       98.7083       1.00000       35.4328  0.000000E+00      0.883702  0.000000E+00      0.883702  0.000000E+00  0.000000E+00      -1.76740       82.1558      0.672497        1       Scatag_D
      31       1733.48       5432.22  0.000000E+00  0.000000E+00       2459.12  0.000000E+00       2197.28       8669.33  0.000000E+00       12125.8       45.9746       6349.85 -1.000000E+10 -1.000000E+10 -1.000000E+10 -1.000000E+10 -1.000000E+10  0.000000E+00  0.000000E+00  0.000000E+00  0.000000E+00  0.000000E+00  0.000000E+00  0.000000E+00  0.000000E+00  0.000000E+00  0.000000E+00  0.000000E+00  0.000000E+00       Funtag_D
      32  3.885626E+05  3.495758E+05       38986.8       17.6670     0.0281557      0.408931      0.188547      0.243196    0.00705999   -0.00733919    0.00784903      0.998141       Opttag_D
      32       80.5965       1.14893       1.00000  0.000000E+00       1.00000  0.000000E+00      0.944162       98.6346       1.00000       35.4195  0.000000E+00      0.878539  0.000000E+00      0.878539  0.000000E+00  0.000000E+00      -1.75708       82.1558      0.672497        1       Scatag_D
      32       1737.22       5440.59  0.000000E+00  0.000000E+00       2459.82  0.000000E+00       2199.57       8620.91  0.000000E+00       12130.4       45.9524       6352.39 -1.000000E+10 -1.000000E+10 -1.000000E+10 -1.000000E+10 -1.000000E+10  0.000000E+00  0.000000E+00  0.000000E+00  0.000000E+00  0.000000E+00  0.000000E+00  0.000000E+00  0.000000E+00  0.000000E+00  0.000000E+00  0.000000E+00  0.000000E+00       Funtag_D
      33  3.885034E+05  3.494916E+05       39011.8       34.5173     0.0300375      0.444162      0.188522      0.243078    0.00730300   -0.00768427    0.00788057       1.00237       Opttag_D
      33       80.6372       1.14550       1.00000  0.000000E+00       1.00000  0.000000E+00      0.944123       98.5124       1.00000       35.3951  0.000000E+00      0.869588  0.000000E+00      0.869588  0.000000E+00  0.000000E+00      -1.73918       82.1558      0.672497        1       Scatag_D
      33       1751.21       5486.78  0.000000E+00  0.000000E+00       2461.23  0.000000E+00       2203.68       8561.68  0.000000E+00       12141.3       46.0657       6359.81 -1.000000E+10 -1.000000E+10 -1.000000E+10 -1.000000E+10 -1.000000E+10  0.000000E+00  0.000000E+00  0.000000E+00  0.000000E+00  0.000000E+00  0.000000E+00  0.000000E+00  0.000000E+00  0.000000E+00  0.000000E+00  0.000000E+00  0.000000E+00       Funtag_D
      34  3.884098E+05  3.494578E+05       38952.0       16.2439     0.0308934      0.459752      0.188513      0.243031    0.00740067   -0.00784293    0.00786507      0.998552       Opttag_D
      34       80.6556       1.14410       1.00000  0.000000E+00       1.00000  0.000000E+00      0.944110       98.4567       1.00000       35.3854  0.000000E+00      0.865936  0.000000E+00      0.865936  0.000000E+00  0.000000E+00      -1.73187       82.1558      0.672497        1       Scatag_D
      34       1744.33       5445.06  0.000000E+00  0.000000E+00       2462.14  0.000000E+00       2205.38       8541.30  0.000000E+00       12146.8       46.1035       6360.86 -1.000000E+10 -1.000000E+10 -1.000000E+10 -1.000000E+10 -1.000000E+10  0.000000E+00  0.000000E+00  0.000000E+00  0.000000E+00  0.000000E+00  0.000000E+00  0.000000E+00  0.000000E+00  0.000000E+00  0.000000E+00  0.000000E+00  0.000000E+00       Funtag_D
      35  3.883488E+05  3.494238E+05       38925.0       12.1716     0.0316316      0.473062      0.188499      0.242981    0.00749885   -0.00797526    0.00786388      0.998619       Opttag_D
      35       80.6735       1.14302       1.00000  0.000000E+00       1.00000  0.000000E+00      0.944106       98.4157       1.00000       35.3750  0.000000E+00      0.863202  0.000000E+00      0.863202  0.000000E+00  0.000000E+00      -1.72640       82.1558      0.672497        1       Scatag_D
      35       1743.80       5445.80  0.000000E+00  0.000000E+00       2463.04  0.000000E+00       2206.01       8505.99  0.000000E+00       12152.0       46.2050       6362.15 -1.000000E+10 -1.000000E+10 -1.000000E+10 -1.000000E+10 -1.000000E+10  0.000000E+00  0.000000E+00  0.000000E+00  0.000000E+00  0.000000E+00  0.000000E+00  0.000000E+00  0.000000E+00  0.000000E+00  0.000000E+00  0.000000E+00  0.000000E+00       Funtag_D
      36  3.882831E+05  3.493705E+05       38912.6       15.9469     0.0328072      0.493882      0.188473      0.242902    0.00765283   -0.00818185    0.00786750      0.999621       Opttag_D
      36       80.7036       1.14146       1.00000  0.000000E+00       1.00000  0.000000E+00      0.944106       98.3537       1.00000       35.3585  0.000000E+00      0.858915  0.000000E+00      0.858915  0.000000E+00  0.000000E+00      -1.71783       82.1558      0.672497        1       Scatag_D
      36       1745.41       5456.73  0.000000E+00  0.000000E+00       2464.49  0.000000E+00       2206.80       8467.64  0.000000E+00       12160.4       46.3904       6364.68 -1.000000E+10 -1.000000E+10 -1.000000E+10 -1.000000E+10 -1.000000E+10  0.000000E+00  0.000000E+00  0.000000E+00  0.000000E+00  0.000000E+00  0.000000E+00  0.000000E+00  0.000000E+00  0.000000E+00  0.000000E+00  0.000000E+00  0.000000E+00       Funtag_D
      37  3.882050E+05  3.492771E+05       38927.9       27.1936     0.0350031      0.531430      0.188424      0.242757    0.00792237   -0.00852367    0.00788858       1.00278       Opttag_D
      37       80.7624       1.13902       1.00000  0.000000E+00       1.00000  0.000000E+00      0.944122       98.2400       1.00000       35.3280  0.000000E+00      0.851251  0.000000E+00      0.851251  0.000000E+00  0.000000E+00      -1.70250       82.1558      0.672497        1       Scatag_D
      37       1754.77       5491.23  0.000000E+00  0.000000E+00       2467.42  0.000000E+00       2207.68       8414.13  0.000000E+00       12176.3       46.7129       6369.61 -1.000000E+10 -1.000000E+10 -1.000000E+10 -1.000000E+10 -1.000000E+10  0.000000E+00  0.000000E+00  0.000000E+00  0.000000E+00  0.000000E+00  0.000000E+00  0.000000E+00  0.000000E+00  0.000000E+00  0.000000E+00  0.000000E+00  0.000000E+00       Funtag_D
      38  3.881175E+05  3.492110E+05       38906.5       15.3807     0.0368258      0.561629      0.188387      0.242644    0.00811344   -0.00877059    0.00788221       1.00116       Opttag_D
      38       80.8126       1.13748       1.00000  0.000000E+00       1.00000  0.000000E+00      0.944140       98.1506       1.00000       35.3052  0.000000E+00      0.845107  0.000000E+00      0.845107  0.000000E+00  0.000000E+00      -1.69021       82.1558      0.672497        1       Scatag_D
      38       1751.94       5473.58  0.000000E+00  0.000000E+00       2469.86  0.000000E+00       2208.57       8391.36  0.000000E+00       12190.5       46.9292       6373.76 -1.000000E+10 -1.000000E+10 -1.000000E+10 -1.000000E+10 -1.000000E+10  0.000000E+00  0.000000E+00  0.000000E+00  0.000000E+00  0.000000E+00  0.000000E+00  0.000000E+00  0.000000E+00  0.000000E+00  0.000000E+00  0.000000E+00  0.000000E+00       Funtag_D
      39  3.880581E+05  3.491775E+05       38880.6       11.7718     0.0375468      0.572962      0.188363      0.242589    0.00821010   -0.00880053    0.00788415       1.00109       Opttag_D
      39       80.8352       1.13693       1.00000  0.000000E+00       1.00000  0.000000E+00      0.944158       98.1177       1.00000       35.2920  0.000000E+00      0.842684  0.000000E+00      0.842684  0.000000E+00  0.000000E+00      -1.68537       82.1558      0.672497        1       Scatag_D
      39       1752.80       5472.80  0.000000E+00  0.000000E+00       2470.94  0.000000E+00       2207.95       8358.44  0.000000E+00       12196.0       46.9231       6374.70 -1.000000E+10 -1.000000E+10 -1.000000E+10 -1.000000E+10 -1.000000E+10  0.000000E+00  0.000000E+00  0.000000E+00  0.000000E+00  0.000000E+00  0.000000E+00  0.000000E+00  0.000000E+00  0.000000E+00  0.000000E+00  0.000000E+00  0.000000E+00       Funtag_D
      40  3.879773E+05  3.491080E+05       38869.3       16.2264     0.0390857      0.596824      0.188311      0.242461    0.00841103   -0.00878641    0.00789266       1.00219       Opttag_D
      40       80.8846       1.13591       1.00000  0.000000E+00       1.00000  0.000000E+00      0.944197       98.0505       1.00000       35.2648  0.000000E+00      0.837521  0.000000E+00      0.837521  0.000000E+00  0.000000E+00      -1.67504       82.1558      0.672497        1       Scatag_D
      40       1756.59       5484.81  0.000000E+00  0.000000E+00       2473.36  0.000000E+00       2206.68       8315.74  0.000000E+00       12208.0       46.8532       6377.25 -1.000000E+10 -1.000000E+10 -1.000000E+10 -1.000000E+10 -1.000000E+10  0.000000E+00  0.000000E+00  0.000000E+00  0.000000E+00  0.000000E+00  0.000000E+00  0.000000E+00  0.000000E+00  0.000000E+00  0.000000E+00  0.000000E+00  0.000000E+00       Funtag_D
      41  3.879291E+05  3.490281E+05       38900.9       23.9180     0.0411696      0.628763      0.188250      0.242302    0.00864154   -0.00870358    0.00791512       1.00423       Opttag_D
      41       80.9506       1.13487       1.00000  0.000000E+00       1.00000  0.000000E+00      0.944241       97.9630       1.00000       35.2352  0.000000E+00      0.830531  0.000000E+00      0.830531  0.000000E+00  0.000000E+00      -1.66106       82.1558      0.672497        1       Scatag_D
      41       1766.60       5507.11  0.000000E+00  0.000000E+00       2476.49  0.000000E+00       2206.32       8289.21  0.000000E+00       12226.3       46.7934       6382.15 -1.000000E+10 -1.000000E+10 -1.000000E+10 -1.000000E+10 -1.000000E+10  0.000000E+00  0.000000E+00  0.000000E+00  0.000000E+00  0.000000E+00  0.000000E+00  0.000000E+00  0.000000E+00  0.000000E+00  0.000000E+00  0.000000E+00  0.000000E+00       Funtag_D
      42  3.878653E+05  3.490087E+05       38856.6       12.9896     0.0415176      0.633759      0.188234      0.242263    0.00869764   -0.00862980    0.00790364       1.00238       Opttag_D
      42       80.9629       1.13461       1.00000  0.000000E+00       1.00000  0.000000E+00      0.944248       97.9518       1.00000       35.2297  0.000000E+00      0.829113  0.000000E+00      0.829113  0.000000E+00  0.000000E+00      -1.65823       82.1558      0.672497        1       Scatag_D
      42       1761.48       5486.89  0.000000E+00  0.000000E+00       2477.12  0.000000E+00       2206.16       8266.50  0.000000E+00       12229.4       46.7877       6382.26 -1.000000E+10 -1.000000E+10 -1.000000E+10 -1.000000E+10 -1.000000E+10  0.000000E+00  0.000000E+00  0.000000E+00  0.000000E+00  0.000000E+00  0.000000E+00  0.000000E+00  0.000000E+00  0.000000E+00  0.000000E+00  0.000000E+00  0.000000E+00       Funtag_D
      43  3.877973E+05  3.489581E+05       38839.2       11.3527     0.0427003      0.650923      0.188196      0.242163    0.00884379   -0.00850677    0.00790254       1.00261       Opttag_D
      43       81.0019       1.13392       1.00000  0.000000E+00       1.00000  0.000000E+00      0.944265       97.9089       1.00000       35.2168  0.000000E+00      0.824665  0.000000E+00      0.824665  0.000000E+00  0.000000E+00      -1.64933       82.1558      0.672497        1       Scatag_D
      43       1760.99       5489.44  0.000000E+00  0.000000E+00       2478.90  0.000000E+00       2206.45       8231.42  0.000000E+00       12240.5       46.8642       6384.65 -1.000000E+10 -1.000000E+10 -1.000000E+10 -1.000000E+10 -1.000000E+10  0.000000E+00  0.000000E+00  0.000000E+00  0.000000E+00  0.000000E+00  0.000000E+00  0.000000E+00  0.000000E+00  0.000000E+00  0.000000E+00  0.000000E+00  0.000000E+00       Funtag_D
      44  3.877469E+05  3.489050E+05       38841.8       13.1502     0.0440428      0.669626      0.188156      0.242068    0.00899696   -0.00841355    0.00790710       1.00291       Opttag_D
      44       81.0463       1.13323       1.00000  0.000000E+00       1.00000  0.000000E+00      0.944286       97.8595       1.00000       35.2055  0.000000E+00      0.819589  0.000000E+00      0.819589  0.000000E+00  0.000000E+00      -1.63918       82.1558      0.672497        1       Scatag_D
      44       1763.02       5492.66  0.000000E+00  0.000000E+00       2480.98  0.000000E+00       2207.15       8210.05  0.000000E+00       12253.3       47.0216       6387.65 -1.000000E+10 -1.000000E+10 -1.000000E+10 -1.000000E+10 -1.000000E+10  0.000000E+00  0.000000E+00  0.000000E+00  0.000000E+00  0.000000E+00  0.000000E+00  0.000000E+00  0.000000E+00  0.000000E+00  0.000000E+00  0.000000E+00  0.000000E+00       Funtag_D
      45  3.876927E+05  3.487761E+05       38916.6       27.0520     0.0477445      0.718725      0.188065      0.241851    0.00936925   -0.00828013    0.00793155       1.00509       Opttag_D
      45       81.1697       1.13181       1.00000  0.000000E+00       1.00000  0.000000E+00      0.944347       97.7247       1.00000       35.1883  0.000000E+00      0.805952  0.000000E+00      0.805952  0.000000E+00  0.000000E+00      -1.61190       82.1558      0.672497        1       Scatag_D
      45       1773.94       5516.59  0.000000E+00  0.000000E+00       2486.46  0.000000E+00       2210.64       8193.76  0.000000E+00       12290.0       47.5876       6397.63 -1.000000E+10 -1.000000E+10 -1.000000E+10 -1.000000E+10 -1.000000E+10  0.000000E+00  0.000000E+00  0.000000E+00  0.000000E+00  0.000000E+00  0.000000E+00  0.000000E+00  0.000000E+00  0.000000E+00  0.000000E+00  0.000000E+00  0.000000E+00       Funtag_D
      46  3.876099E+05  3.487480E+05       38861.9       11.5054     0.0485420      0.728235      0.188048      0.241818    0.00945035   -0.00830066    0.00792587       1.00324       Opttag_D
      46       81.1972       1.13145       1.00000  0.000000E+00       1.00000  0.000000E+00      0.944360       97.6948       1.00000       35.1888  0.000000E+00      0.802647  0.000000E+00      0.802647  0.000000E+00  0.000000E+00      -1.60529       82.1558      0.672497        1       Scatag_D
      46       1771.40       5496.26  0.000000E+00  0.000000E+00       2487.87  0.000000E+00       2211.68       8150.47  0.000000E+00       12298.0       47.7142       6398.46 -1.000000E+10 -1.000000E+10 -1.000000E+10 -1.000000E+10 -1.000000E+10  0.000000E+00  0.000000E+00  0.000000E+00  0.000000E+00  0.000000E+00  0.000000E+00  0.000000E+00  0.000000E+00  0.000000E+00  0.000000E+00  0.000000E+00  0.000000E+00       Funtag_D
      47  3.875659E+05  3.487286E+05       38837.3       10.0693     0.0489306      0.731958      0.188036      0.241817    0.00950656   -0.00838047    0.00792734       1.00301       Opttag_D
      47       81.2118       1.13110       1.00000  0.000000E+00       1.00000  0.000000E+00      0.944368       97.6792       1.00000       35.1919  0.000000E+00      0.800776  0.000000E+00      0.800776  0.000000E+00  0.000000E+00      -1.60155       82.1558      0.672497        1       Scatag_D
      47       1772.06       5493.74  0.000000E+00  0.000000E+00       2488.59  0.000000E+00       2212.38       8123.15  0.000000E+00       12301.4       47.7798       6398.29 -1.000000E+10 -1.000000E+10 -1.000000E+10 -1.000000E+10 -1.000000E+10  0.000000E+00  0.000000E+00  0.000000E+00  0.000000E+00  0.000000E+00  0.000000E+00  0.000000E+00  0.000000E+00  0.000000E+00  0.000000E+00  0.000000E+00  0.000000E+00       Funtag_D
      48  3.875113E+05  3.486795E+05       38831.9       12.3775     0.0502278      0.746465      0.188006      0.241789    0.00964834   -0.00851365    0.00793373       1.00304       Opttag_D
      48       81.2576       1.13040       1.00000  0.000000E+00       1.00000  0.000000E+00      0.944391       97.6288       1.00000       35.1979  0.000000E+00      0.795430  0.000000E+00      0.795430  0.000000E+00  0.000000E+00      -1.59086       82.1558      0.672497        1       Scatag_D
      48       1774.92       5494.16  0.000000E+00  0.000000E+00       2490.78  0.000000E+00       2214.44       8096.20  0.000000E+00       12313.6       47.8855       6399.90 -1.000000E+10 -1.000000E+10 -1.000000E+10 -1.000000E+10 -1.000000E+10  0.000000E+00  0.000000E+00  0.000000E+00  0.000000E+00  0.000000E+00  0.000000E+00  0.000000E+00  0.000000E+00  0.000000E+00  0.000000E+00  0.000000E+00  0.000000E+00       Funtag_D
      49  3.874801E+05  3.485842E+05       38895.9       28.5896     0.0530677      0.777747      0.187939      0.241726    0.00992346   -0.00870020    0.00795693       1.00471       Opttag_D
      49       81.3558       1.12919       1.00000  0.000000E+00       1.00000  0.000000E+00      0.944441       97.5168       1.00000       35.2145  0.000000E+00      0.784092  0.000000E+00      0.784092  0.000000E+00  0.000000E+00      -1.56818       82.1558      0.672497        1       Scatag_D
      49       1785.31       5512.38  0.000000E+00  0.000000E+00       2495.64  0.000000E+00       2218.75       8089.62  0.000000E+00       12340.8       47.9691       6405.44 -1.000000E+10 -1.000000E+10 -1.000000E+10 -1.000000E+10 -1.000000E+10  0.000000E+00  0.000000E+00  0.000000E+00  0.000000E+00  0.000000E+00  0.000000E+00  0.000000E+00  0.000000E+00  0.000000E+00  0.000000E+00  0.000000E+00  0.000000E+00       Funtag_D
      50  3.874076E+05  3.485670E+05       38840.6       12.1905     0.0536298      0.783665      0.187927      0.241714    0.00997306   -0.00872845    0.00794928       1.00326       Opttag_D
      50       81.3748       1.12898       1.00000  0.000000E+00       1.00000  0.000000E+00      0.944450       97.4952       1.00000       35.2182  0.000000E+00      0.782003  0.000000E+00      0.782003  0.000000E+00  0.000000E+00      -1.56401       82.1558      0.672497        1       Scatag_D
      50       1781.88       5496.48  0.000000E+00  0.000000E+00       2496.60  0.000000E+00       2219.50       8046.13  0.000000E+00       12346.1       47.9250       6405.99 -1.000000E+10 -1.000000E+10 -1.000000E+10 -1.000000E+10 -1.000000E+10  0.000000E+00  0.000000E+00  0.000000E+00  0.000000E+00  0.000000E+00  0.000000E+00  0.000000E+00  0.000000E+00  0.000000E+00  0.000000E+00  0.000000E+00  0.000000E+00       Funtag_D
      51  3.873634E+05  3.485357E+05       38827.7       9.23148     0.0546266      0.794009      0.187902      0.241686     0.0100636   -0.00874765    0.00795000       1.00336       Opttag_D
      51       81.4083       1.12858       1.00000  0.000000E+00       1.00000  0.000000E+00      0.944466       97.4554       1.00000       35.2248  0.000000E+00      0.778216  0.000000E+00      0.778216  0.000000E+00  0.000000E+00      -1.55643       82.1558      0.672497        1       Scatag_D
      51       1782.20       5497.64  0.000000E+00  0.000000E+00       2498.24  0.000000E+00       2220.63       8018.08  0.000000E+00       12355.3       47.8719       6407.75 -1.000000E+10 -1.000000E+10 -1.000000E+10 -1.000000E+10 -1.000000E+10  0.000000E+00  0.000000E+00  0.000000E+00  0.000000E+00  0.000000E+00  0.000000E+00  0.000000E+00  0.000000E+00  0.000000E+00  0.000000E+00  0.000000E+00  0.000000E+00       Funtag_D
      52  3.873160E+05  3.484812E+05       38834.8       11.5739     0.0564283      0.811839      0.187860      0.241638     0.0102210   -0.00878858    0.00795595       1.00380       Opttag_D
      52       81.4690       1.12789       1.00000  0.000000E+00       1.00000  0.000000E+00      0.944497       97.3838       1.00000       35.2396  0.000000E+00      0.771241  0.000000E+00      0.771241  0.000000E+00  0.000000E+00      -1.54248       82.1558      0.672497        1       Scatag_D
      52       1784.87       5502.48  0.000000E+00  0.000000E+00       2501.28  0.000000E+00       2222.87       7991.98  0.000000E+00       12372.0       47.8703       6411.48 -1.000000E+10 -1.000000E+10 -1.000000E+10 -1.000000E+10 -1.000000E+10  0.000000E+00  0.000000E+00  0.000000E+00  0.000000E+00  0.000000E+00  0.000000E+00  0.000000E+00  0.000000E+00  0.000000E+00  0.000000E+00  0.000000E+00  0.000000E+00       Funtag_D
      53  3.872593E+05  3.483900E+05       38869.3       20.9874     0.0594656      0.838930      0.187791      0.241572     0.0104842   -0.00886489    0.00796588       1.00507       Opttag_D
      53       81.5705       1.12666       1.00000  0.000000E+00       1.00000  0.000000E+00      0.944549       97.2639       1.00000       35.2698  0.000000E+00      0.759683  0.000000E+00      0.759683  0.000000E+00  0.000000E+00      -1.51937       82.1558      0.672497        1       Scatag_D
      53       1789.33       5516.38  0.000000E+00  0.000000E+00       2506.16  0.000000E+00       2226.74       7965.64  0.000000E+00       12398.7       48.0515       6418.31 -1.000000E+10 -1.000000E+10 -1.000000E+10 -1.000000E+10 -1.000000E+10  0.000000E+00  0.000000E+00  0.000000E+00  0.000000E+00  0.000000E+00  0.000000E+00  0.000000E+00  0.000000E+00  0.000000E+00  0.000000E+00  0.000000E+00  0.000000E+00       Funtag_D
      54  3.871989E+05  3.483225E+05       38876.4       12.5047     0.0620965      0.861547      0.187739      0.241531     0.0106791   -0.00893516    0.00797651       1.00481       Opttag_D
      54       81.6580       1.12590       1.00000  0.000000E+00       1.00000  0.000000E+00      0.944596       97.1624       1.00000       35.3015  0.000000E+00      0.750121  0.000000E+00      0.750121  0.000000E+00  0.000000E+00      -1.50024       82.1558      0.672497        1       Scatag_D
      54       1794.11       5513.54  0.000000E+00  0.000000E+00       2510.66  0.000000E+00       2230.41       7932.50  0.000000E+00       12422.5       48.2477       6424.47 -1.000000E+10 -1.000000E+10 -1.000000E+10 -1.000000E+10 -1.000000E+10  0.000000E+00  0.000000E+00  0.000000E+00  0.000000E+00  0.000000E+00  0.000000E+00  0.000000E+00  0.000000E+00  0.000000E+00  0.000000E+00  0.000000E+00  0.000000E+00       Funtag_D
      55  3.871598E+05  3.482951E+05       38864.6       8.80296     0.0627822      0.865685      0.187720      0.241520     0.0107582   -0.00895967    0.00797561       1.00480       Opttag_D
      55       81.6806       1.12536       1.00000  0.000000E+00       1.00000  0.000000E+00      0.944610       97.1348       1.00000       35.3107  0.000000E+00      0.747842  0.000000E+00      0.747842  0.000000E+00  0.000000E+00      -1.49568       82.1558      0.672497        1       Scatag_D
      55       1793.70       5513.39  0.000000E+00  0.000000E+00       2511.75  0.000000E+00       2231.13       7913.84  0.000000E+00       12426.9       48.3366       6425.61 -1.000000E+10 -1.000000E+10 -1.000000E+10 -1.000000E+10 -1.000000E+10  0.000000E+00  0.000000E+00  0.000000E+00  0.000000E+00  0.000000E+00  0.000000E+00  0.000000E+00  0.000000E+00  0.000000E+00  0.000000E+00  0.000000E+00  0.000000E+00       Funtag_D
      56  3.871132E+05  3.482473E+05       38865.9       11.5904     0.0638903      0.871295      0.187687      0.241509     0.0108963   -0.00901920    0.00797798       1.00501       Opttag_D
      56       81.7168       1.12437       1.00000  0.000000E+00       1.00000  0.000000E+00      0.944634       97.0904       1.00000       35.3256  0.000000E+00      0.744619  0.000000E+00      0.744619  0.000000E+00  0.000000E+00      -1.48924       82.1558      0.672497        1       Scatag_D
      56       1794.77       5515.73  0.000000E+00  0.000000E+00       2513.38  0.000000E+00       2232.38       7900.60  0.000000E+00       12433.2       48.4459       6427.43 -1.000000E+10 -1.000000E+10 -1.000000E+10 -1.000000E+10 -1.000000E+10  0.000000E+00  0.000000E+00  0.000000E+00  0.000000E+00  0.000000E+00  0.000000E+00  0.000000E+00  0.000000E+00  0.000000E+00  0.000000E+00  0.000000E+00  0.000000E+00       Funtag_D
      57  3.870731E+05  3.481851E+05       38887.9       15.2242     0.0656978      0.881307      0.187642      0.241494     0.0110758   -0.00909708    0.00798195       1.00549       Opttag_D
      57       81.7756       1.12327       1.00000  0.000000E+00       1.00000  0.000000E+00      0.944671       97.0194       1.00000       35.3511  0.000000E+00      0.739831  0.000000E+00      0.739831  0.000000E+00  0.000000E+00      -1.47966       82.1558      0.672497        1       Scatag_D
      57       1796.56       5521.02  0.000000E+00  0.000000E+00       2516.26  0.000000E+00       2234.94       7894.58  0.000000E+00       12445.3       48.4626       6430.75 -1.000000E+10 -1.000000E+10 -1.000000E+10 -1.000000E+10 -1.000000E+10  0.000000E+00  0.000000E+00  0.000000E+00  0.000000E+00  0.000000E+00  0.000000E+00  0.000000E+00  0.000000E+00  0.000000E+00  0.000000E+00  0.000000E+00  0.000000E+00       Funtag_D
      58  3.870257E+05  3.481507E+05       38875.0       9.89926     0.0666976      0.885657      0.187616      0.241491     0.0111752   -0.00913861    0.00797954       1.00530       Opttag_D
      58       81.8077       1.12263       1.00000  0.000000E+00       1.00000  0.000000E+00      0.944693       96.9778       1.00000       35.3655  0.000000E+00      0.737680  0.000000E+00      0.737680  0.000000E+00  0.000000E+00      -1.47536       82.1558      0.672497        1       Scatag_D
      58       1795.47       5518.88  0.000000E+00  0.000000E+00       2517.76  0.000000E+00       2236.53       7874.21  0.000000E+00       12451.8       48.3473       6431.95 -1.000000E+10 -1.000000E+10 -1.000000E+10 -1.000000E+10 -1.000000E+10  0.000000E+00  0.000000E+00  0.000000E+00  0.000000E+00  0.000000E+00  0.000000E+00  0.000000E+00  0.000000E+00  0.000000E+00  0.000000E+00  0.000000E+00  0.000000E+00       Funtag_D
      59  3.869678E+05  3.481016E+05       38866.2       10.0160     0.0683709      0.892236      0.187583      0.241490     0.0113172   -0.00917677    0.00797662       1.00546       Opttag_D
      59       81.8610       1.12183       1.00000  0.000000E+00       1.00000  0.000000E+00      0.944731       96.9060       1.00000       35.3914  0.000000E+00      0.734105  0.000000E+00      0.734105  0.000000E+00  0.000000E+00      -1.46821       82.1558      0.672497        1       Scatag_D
      59       1794.16       5520.62  0.000000E+00  0.000000E+00       2520.35  0.000000E+00       2239.50       7846.54  0.000000E+00       12463.3       48.0632       6433.71 -1.000000E+10 -1.000000E+10 -1.000000E+10 -1.000000E+10 -1.000000E+10  0.000000E+00  0.000000E+00  0.000000E+00  0.000000E+00  0.000000E+00  0.000000E+00  0.000000E+00  0.000000E+00  0.000000E+00  0.000000E+00  0.000000E+00  0.000000E+00       Funtag_D
      60  3.869273E+05  3.480590E+05       38868.3       14.3002     0.0701316      0.897708      0.187553      0.241485     0.0114402   -0.00919645    0.00797502       1.00582       Opttag_D
      60       81.9166       1.12116       1.00000  0.000000E+00       1.00000  0.000000E+00      0.944775       96.8253       1.00000       35.4219  0.000000E+00      0.729853  0.000000E+00      0.729853  0.000000E+00  0.000000E+00      -1.45971       82.1558      0.672497        1       Scatag_D
      60       1793.44       5524.64  0.000000E+00  0.000000E+00       2523.16  0.000000E+00       2242.93       7826.00  0.000000E+00       12475.3       47.7950       6434.98 -1.000000E+10 -1.000000E+10 -1.000000E+10 -1.000000E+10 -1.000000E+10  0.000000E+00  0.000000E+00  0.000000E+00  0.000000E+00  0.000000E+00  0.000000E+00  0.000000E+00  0.000000E+00  0.000000E+00  0.000000E+00  0.000000E+00  0.000000E+00       Funtag_D
      61  3.868813E+05  3.480264E+05       38854.9       10.0253     0.0715902      0.901117      0.187531      0.241484     0.0115343   -0.00918993    0.00797134       1.00578       Opttag_D
      61       81.9622       1.12065       1.00000  0.000000E+00       1.00000  0.000000E+00      0.944814       96.7543       1.00000       35.4496  0.000000E+00      0.726146  0.000000E+00      0.726146  0.000000E+00  0.000000E+00      -1.45229       82.1558      0.672497        1       Scatag_D
      61       1791.78       5524.13  0.000000E+00  0.000000E+00       2525.46  0.000000E+00       2245.99       7799.54  0.000000E+00       12485.0       47.6152       6435.43 -1.000000E+10 -1.000000E+10 -1.000000E+10 -1.000000E+10 -1.000000E+10  0.000000E+00  0.000000E+00  0.000000E+00  0.000000E+00  0.000000E+00  0.000000E+00  0.000000E+00  0.000000E+00  0.000000E+00  0.000000E+00  0.000000E+00  0.000000E+00       Funtag_D
      62  3.868336E+05  3.479934E+05       38840.2       9.81134     0.0732419      0.903435      0.187511      0.241479     0.0116294   -0.00914839    0.00797085       1.00595       Opttag_D
      62       82.0131       1.12012       1.00000  0.000000E+00       1.00000  0.000000E+00      0.944860       96.6697       1.00000       35.4820  0.000000E+00      0.721330  0.000000E+00      0.721330  0.000000E+00  0.000000E+00      -1.44266       82.1558      0.672497        1       Scatag_D
      62       1791.56       5526.09  0.000000E+00  0.000000E+00       2527.99  0.000000E+00       2249.52       7767.86  0.000000E+00       12495.0       47.4975       6434.66 -1.000000E+10 -1.000000E+10 -1.000000E+10 -1.000000E+10 -1.000000E+10  0.000000E+00  0.000000E+00  0.000000E+00  0.000000E+00  0.000000E+00  0.000000E+00  0.000000E+00  0.000000E+00  0.000000E+00  0.000000E+00  0.000000E+00  0.000000E+00       Funtag_D
      63  3.867955E+05  3.479596E+05       38835.9       11.7044     0.0747963      0.904563      0.187489      0.241481     0.0117272   -0.00909152    0.00797385       1.00608       Opttag_D
      63       82.0602       1.11946       1.00000  0.000000E+00       1.00000  0.000000E+00      0.944906       96.5861       1.00000       35.5129  0.000000E+00      0.716840  0.000000E+00      0.716840  0.000000E+00  0.000000E+00      -1.43368       82.1558      0.672497        1       Scatag_D
      63       1792.91       5527.46  0.000000E+00  0.000000E+00       2530.37  0.000000E+00       2252.71       7748.66  0.000000E+00       12503.7       47.4658       6432.59 -1.000000E+10 -1.000000E+10 -1.000000E+10 -1.000000E+10 -1.000000E+10  0.000000E+00  0.000000E+00  0.000000E+00  0.000000E+00  0.000000E+00  0.000000E+00  0.000000E+00  0.000000E+00  0.000000E+00  0.000000E+00  0.000000E+00  0.000000E+00       Funtag_D
      64  3.867580E+05  3.479354E+05       38822.6       9.32335     0.0758388      0.904431      0.187471      0.241474     0.0117970   -0.00902246    0.00797822       1.00616       Opttag_D
      64       82.0913       1.11894       1.00000  0.000000E+00       1.00000  0.000000E+00      0.944938       96.5296       1.00000       35.5319  0.000000E+00      0.713823  0.000000E+00      0.713823  0.000000E+00  0.000000E+00      -1.42765       82.1558      0.672497        1       Scatag_D
      64       1794.88       5528.32  0.000000E+00  0.000000E+00       2531.91  0.000000E+00       2254.61       7726.45  0.000000E+00       12509.0       47.4976       6429.96 -1.000000E+10 -1.000000E+10 -1.000000E+10 -1.000000E+10 -1.000000E+10  0.000000E+00  0.000000E+00  0.000000E+00  0.000000E+00  0.000000E+00  0.000000E+00  0.000000E+00  0.000000E+00  0.000000E+00  0.000000E+00  0.000000E+00  0.000000E+00       Funtag_D
      65  3.867079E+05  3.478965E+05       38811.4       10.1044     0.0774632      0.903117      0.187442      0.241465     0.0119093   -0.00888119    0.00798624       1.00625       Opttag_D
      65       82.1393       1.11814       1.00000  0.000000E+00       1.00000  0.000000E+00      0.944986       96.4440       1.00000       35.5595  0.000000E+00      0.709625  0.000000E+00      0.709625  0.000000E+00  0.000000E+00      -1.41925       82.1558      0.672497        1       Scatag_D
      65       1798.49       5529.37  0.000000E+00  0.000000E+00       2534.32  0.000000E+00       2257.15       7703.20  0.000000E+00       12517.1       47.5696       6424.18 -1.000000E+10 -1.000000E+10 -1.000000E+10 -1.000000E+10 -1.000000E+10  0.000000E+00  0.000000E+00  0.000000E+00  0.000000E+00  0.000000E+00  0.000000E+00  0.000000E+00  0.000000E+00  0.000000E+00  0.000000E+00  0.000000E+00  0.000000E+00       Funtag_D
      66  3.866651E+05  3.478559E+05       38809.3       13.9039     0.0793903      0.901176      0.187413      0.241465     0.0120267   -0.00872471    0.00799298       1.00643       Opttag_D
      66       82.1958       1.11736       1.00000  0.000000E+00       1.00000  0.000000E+00      0.945042       96.3449       1.00000       35.5943  0.000000E+00      0.705442  0.000000E+00      0.705442  0.000000E+00  0.000000E+00      -1.41088       82.1558      0.672497        1       Scatag_D
      66       1801.53       5531.37  0.000000E+00  0.000000E+00       2537.45  0.000000E+00       2259.91       7686.37  0.000000E+00       12528.2       47.5571       6416.84 -1.000000E+10 -1.000000E+10 -1.000000E+10 -1.000000E+10 -1.000000E+10  0.000000E+00  0.000000E+00  0.000000E+00  0.000000E+00  0.000000E+00  0.000000E+00  0.000000E+00  0.000000E+00  0.000000E+00  0.000000E+00  0.000000E+00  0.000000E+00       Funtag_D
      67  3.866197E+05  3.478299E+05       38789.9       9.99959     0.0808240      0.898919      0.187394      0.241466     0.0121018   -0.00860807    0.00799621       1.00654       Opttag_D
      67       82.2375       1.11696       1.00000  0.000000E+00       1.00000  0.000000E+00      0.945082       96.2744       1.00000       35.6199  0.000000E+00      0.702785  0.000000E+00      0.702785  0.000000E+00  0.000000E+00      -1.40557       82.1558      0.672497        1       Scatag_D
      67       1802.98       5532.53  0.000000E+00  0.000000E+00       2539.85  0.000000E+00       2261.53       7657.58  0.000000E+00       12537.0       47.4980       6410.91 -1.000000E+10 -1.000000E+10 -1.000000E+10 -1.000000E+10 -1.000000E+10  0.000000E+00  0.000000E+00  0.000000E+00  0.000000E+00  0.000000E+00  0.000000E+00  0.000000E+00  0.000000E+00  0.000000E+00  0.000000E+00  0.000000E+00  0.000000E+00       Funtag_D
      68  3.865738E+05  3.478037E+05       38770.1       8.79030     0.0825812      0.895428      0.187377      0.241478     0.0121774   -0.00850773    0.00799507       1.00667       Opttag_D
      68       82.2885       1.11663       1.00000  0.000000E+00       1.00000  0.000000E+00      0.945132       96.1890       1.00000       35.6533  0.000000E+00      0.699945  0.000000E+00      0.699945  0.000000E+00  0.000000E+00      -1.39989       82.1558      0.672497        1       Scatag_D
      68       1802.47       5533.98  0.000000E+00  0.000000E+00       2542.98  0.000000E+00       2262.72       7627.92  0.000000E+00       12548.4       47.3480       6404.26 -1.000000E+10 -1.000000E+10 -1.000000E+10 -1.000000E+10 -1.000000E+10  0.000000E+00  0.000000E+00  0.000000E+00  0.000000E+00  0.000000E+00  0.000000E+00  0.000000E+00  0.000000E+00  0.000000E+00  0.000000E+00  0.000000E+00  0.000000E+00       Funtag_D
      69  3.865420E+05  3.477836E+05       38758.4       11.3715     0.0839837      0.891939      0.187366      0.241492     0.0122354   -0.00851377    0.00799352       1.00678       Opttag_D
      69       82.3287       1.11637       1.00000  0.000000E+00       1.00000  0.000000E+00      0.945172       96.1211       1.00000       35.6812  0.000000E+00      0.697524  0.000000E+00      0.697524  0.000000E+00  0.000000E+00      -1.39505       82.1558      0.672497        1       Scatag_D
      69       1801.77       5535.18  0.000000E+00  0.000000E+00       2545.64  0.000000E+00       2262.94       7607.79  0.000000E+00       12557.6       47.2708       6400.21 -1.000000E+10 -1.000000E+10 -1.000000E+10 -1.000000E+10 -1.000000E+10  0.000000E+00  0.000000E+00  0.000000E+00  0.000000E+00  0.000000E+00  0.000000E+00  0.000000E+00  0.000000E+00  0.000000E+00  0.000000E+00  0.000000E+00  0.000000E+00       Funtag_D
      70  3.865099E+05  3.477697E+05       38740.1       8.34153     0.0849054      0.888927      0.187358      0.241495     0.0122754   -0.00855410    0.00799197       1.00684       Opttag_D
      70       82.3549       1.11618       1.00000  0.000000E+00       1.00000  0.000000E+00      0.945199       96.0769       1.00000       35.6985  0.000000E+00      0.695999  0.000000E+00      0.695999  0.000000E+00  0.000000E+00      -1.39200       82.1558      0.672497        1       Scatag_D
      70       1801.07       5535.83  0.000000E+00  0.000000E+00       2547.45  0.000000E+00       2262.51       7584.77  0.000000E+00       12563.2       47.2445       6398.02 -1.000000E+10 -1.000000E+10 -1.000000E+10 -1.000000E+10 -1.000000E+10  0.000000E+00  0.000000E+00  0.000000E+00  0.000000E+00  0.000000E+00  0.000000E+00  0.000000E+00  0.000000E+00  0.000000E+00  0.000000E+00  0.000000E+00  0.000000E+00       Funtag_D
      71  3.864751E+05  3.477469E+05       38728.2       7.98528     0.0861564      0.885573      0.187344      0.241492     0.0123414   -0.00865637    0.00799246       1.00697       Opttag_D
      71       82.3902       1.11582       1.00000  0.000000E+00       1.00000  0.000000E+00      0.945238       96.0196       1.00000       35.7203  0.000000E+00      0.693693  0.000000E+00      0.693693  0.000000E+00  0.000000E+00      -1.38739       82.1558      0.672497        1       Scatag_D
      71       1801.29       5537.24  0.000000E+00  0.000000E+00       2549.92  0.000000E+00       2261.71       7564.31  0.000000E+00       12570.4       47.2936       6396.03 -1.000000E+10 -1.000000E+10 -1.000000E+10 -1.000000E+10 -1.000000E+10  0.000000E+00  0.000000E+00  0.000000E+00  0.000000E+00  0.000000E+00  0.000000E+00  0.000000E+00  0.000000E+00  0.000000E+00  0.000000E+00  0.000000E+00  0.000000E+00       Funtag_D
      72  3.864475E+05  3.477172E+05       38730.4       11.0146     0.0876465      0.900975      0.187321      0.241480     0.0124272   -0.00874028    0.00799671       1.00710       Opttag_D
      72       82.4319       1.11544       1.00000  0.000000E+00       1.00000  0.000000E+00      0.945282       95.9580       1.00000       35.7438  0.000000E+00      0.691253  0.000000E+00      0.691253  0.000000E+00  0.000000E+00      -1.38251       82.1558      0.672497        1       Scatag_D
      72       1803.21       5538.72  0.000000E+00  0.000000E+00       2552.76  0.000000E+00       2261.55       7552.05  0.000000E+00       12579.0       47.3761       6395.70 -1.000000E+10 -1.000000E+10 -1.000000E+10 -1.000000E+10 -1.000000E+10  0.000000E+00  0.000000E+00  0.000000E+00  0.000000E+00  0.000000E+00  0.000000E+00  0.000000E+00  0.000000E+00  0.000000E+00  0.000000E+00  0.000000E+00  0.000000E+00       Funtag_D
      73  3.864175E+05  3.476921E+05       38725.4       8.08250     0.0888319      0.913125      0.187301      0.241460     0.0124997   -0.00879727    0.00800115       1.00719       Opttag_D
      73       82.4650       1.11518       1.00000  0.000000E+00       1.00000  0.000000E+00      0.945317       95.9146       1.00000       35.7601  0.000000E+00      0.689209  0.000000E+00      0.689209  0.000000E+00  0.000000E+00      -1.37842       82.1558      0.672497        1       Scatag_D
      73       1805.21       5539.71  0.000000E+00  0.000000E+00       2554.91  0.000000E+00       2261.67       7535.03  0.000000E+00       12585.6       47.4446       6395.84 -1.000000E+10 -1.000000E+10 -1.000000E+10 -1.000000E+10 -1.000000E+10  0.000000E+00  0.000000E+00  0.000000E+00  0.000000E+00  0.000000E+00  0.000000E+00  0.000000E+00  0.000000E+00  0.000000E+00  0.000000E+00  0.000000E+00  0.000000E+00       Funtag_D
      74  3.863820E+05  3.476528E+05       38729.2       7.74106     0.0905853      0.930927      0.187268      0.241428     0.0126130   -0.00885571    0.00800718       1.00733       Opttag_D
      74       82.5138       1.11483       1.00000  0.000000E+00       1.00000  0.000000E+00      0.945369       95.8566       1.00000       35.7832  0.000000E+00      0.686373  0.000000E+00      0.686373  0.000000E+00  0.000000E+00      -1.37275       82.1558      0.672497        1       Scatag_D
      74       1807.93       5541.22  0.000000E+00  0.000000E+00       2558.00  0.000000E+00       2262.44       7520.48  0.000000E+00       12595.4       47.4916       6396.24 -1.000000E+10 -1.000000E+10 -1.000000E+10 -1.000000E+10 -1.000000E+10  0.000000E+00  0.000000E+00  0.000000E+00  0.000000E+00  0.000000E+00  0.000000E+00  0.000000E+00  0.000000E+00  0.000000E+00  0.000000E+00  0.000000E+00  0.000000E+00       Funtag_D
      75  3.863537E+05  3.476127E+05       38741.0       11.5433     0.0925764      0.950854      0.187236      0.241400     0.0127288   -0.00893468    0.00801232       1.00751       Opttag_D
      75       82.5692       1.11465       1.00000  0.000000E+00       1.00000  0.000000E+00      0.945426       95.7964       1.00000       35.8112  0.000000E+00      0.683300  0.000000E+00      0.683300  0.000000E+00  0.000000E+00      -1.36660       82.1558      0.672497        1       Scatag_D
      75       1810.25       5543.22  0.000000E+00  0.000000E+00       2561.41  0.000000E+00       2263.60       7511.22  0.000000E+00       12607.0       47.4078       6396.93 -1.000000E+10 -1.000000E+10 -1.000000E+10 -1.000000E+10 -1.000000E+10  0.000000E+00  0.000000E+00  0.000000E+00  0.000000E+00  0.000000E+00  0.000000E+00  0.000000E+00  0.000000E+00  0.000000E+00  0.000000E+00  0.000000E+00  0.000000E+00       Funtag_D
      76  3.863236E+05  3.475849E+05       38738.7       8.08853     0.0938378      0.963365      0.187215      0.241387     0.0128093   -0.00899674    0.00801457       1.00760       Opttag_D
      76       82.6044       1.11454       1.00000  0.000000E+00       1.00000  0.000000E+00      0.945462       95.7620       1.00000       35.8284  0.000000E+00      0.681498  0.000000E+00      0.681498  0.000000E+00  0.000000E+00      -1.36300       82.1558      0.672497        1       Scatag_D
      76       1811.27       5544.19  0.000000E+00  0.000000E+00       2563.46  0.000000E+00       2264.47       7497.72  0.000000E+00       12613.9       47.2697       6396.44 -1.000000E+10 -1.000000E+10 -1.000000E+10 -1.000000E+10 -1.000000E+10  0.000000E+00  0.000000E+00  0.000000E+00  0.000000E+00  0.000000E+00  0.000000E+00  0.000000E+00  0.000000E+00  0.000000E+00  0.000000E+00  0.000000E+00  0.000000E+00       Funtag_D
      77  3.862904E+05  3.475469E+05       38743.5       7.40212     0.0953537      0.978290      0.187189      0.241389     0.0129189   -0.00914115    0.00801630       1.00775       Opttag_D
      77       82.6466       1.11430       1.00000  0.000000E+00       1.00000  0.000000E+00      0.945506       95.7225       1.00000       35.8499  0.000000E+00      0.679388  0.000000E+00      0.679388  0.000000E+00  0.000000E+00      -1.35878       82.1558      0.672497        1       Scatag_D
      77       1812.05       5545.82  0.000000E+00  0.000000E+00       2565.86  0.000000E+00       2265.28       7490.95  0.000000E+00       12621.9       47.0293       6394.64 -1.000000E+10 -1.000000E+10 -1.000000E+10 -1.000000E+10 -1.000000E+10  0.000000E+00  0.000000E+00  0.000000E+00  0.000000E+00  0.000000E+00  0.000000E+00  0.000000E+00  0.000000E+00  0.000000E+00  0.000000E+00  0.000000E+00  0.000000E+00       Funtag_D
      78  3.862621E+05  3.475043E+05       38757.9       10.5704     0.0970884      0.995148      0.187159      0.241397     0.0130420   -0.00936186    0.00801985       1.00795       Opttag_D
      78       82.6945       1.11402       1.00000  0.000000E+00       1.00000  0.000000E+00      0.945555       95.6777       1.00000       35.8755  0.000000E+00      0.677025  0.000000E+00      0.677025  0.000000E+00  0.000000E+00      -1.35405       82.1558      0.672497        1       Scatag_D
      78       1813.66       5548.02  0.000000E+00  0.000000E+00       2568.48  0.000000E+00       2265.86       7491.58  0.000000E+00       12630.8       46.8281       6392.64 -1.000000E+10 -1.000000E+10 -1.000000E+10 -1.000000E+10 -1.000000E+10  0.000000E+00  0.000000E+00  0.000000E+00  0.000000E+00  0.000000E+00  0.000000E+00  0.000000E+00  0.000000E+00  0.000000E+00  0.000000E+00  0.000000E+00  0.000000E+00       Funtag_D
      79  3.862298E+05  3.474621E+05       38767.7       8.59341     0.0986970       1.01059      0.187130      0.241411     0.0131637   -0.00962073    0.00802396       1.00818       Opttag_D
      79       82.7384       1.11369       1.00000  0.000000E+00       1.00000  0.000000E+00      0.945600       95.6362       1.00000       35.8991  0.000000E+00      0.674777  0.000000E+00      0.674777  0.000000E+00  0.000000E+00      -1.34955       82.1558      0.672497        1       Scatag_D
      79       1815.52       5550.61  0.000000E+00  0.000000E+00       2570.78  0.000000E+00       2266.18       7488.47  0.000000E+00       12638.6       46.6839       6390.91 -1.000000E+10 -1.000000E+10 -1.000000E+10 -1.000000E+10 -1.000000E+10  0.000000E+00  0.000000E+00  0.000000E+00  0.000000E+00  0.000000E+00  0.000000E+00  0.000000E+00  0.000000E+00  0.000000E+00  0.000000E+00  0.000000E+00  0.000000E+00       Funtag_D
      80  3.861943E+05  3.474120E+05       38782.3       8.87538      0.100524       1.02790      0.187097      0.241420     0.0133085   -0.00993599    0.00802890       1.00846       Opttag_D
      80       82.7874       1.11320       1.00000  0.000000E+00       1.00000  0.000000E+00      0.945650       95.5888       1.00000       35.9254  0.000000E+00      0.672108  0.000000E+00      0.672108  0.000000E+00  0.000000E+00      -1.34422       82.1558      0.672497        1       Scatag_D
      80       1817.75       5553.62  0.000000E+00  0.000000E+00       2573.30  0.000000E+00       2266.22       7488.51  0.000000E+00       12646.8       46.6499       6389.46 -1.000000E+10 -1.000000E+10 -1.000000E+10 -1.000000E+10 -1.000000E+10  0.000000E+00  0.000000E+00  0.000000E+00  0.000000E+00  0.000000E+00  0.000000E+00  0.000000E+00  0.000000E+00  0.000000E+00  0.000000E+00  0.000000E+00  0.000000E+00       Funtag_D
      81  3.861629E+05  3.473638E+05       38799.1       10.2085      0.102412       1.04552      0.187061      0.241414     0.0134477    -0.0102043    0.00803312       1.00872       Opttag_D
      81       82.8373       1.11282       1.00000  0.000000E+00       1.00000  0.000000E+00      0.945701       95.5427       1.00000       35.9507  0.000000E+00      0.669250  0.000000E+00      0.669250  0.000000E+00  0.000000E+00      -1.33850       82.1558      0.672497        1       Scatag_D
      81       1819.67       5556.53  0.000000E+00  0.000000E+00       2575.75  0.000000E+00       2266.51       7489.72  0.000000E+00       12655.1       46.6715       6389.16 -1.000000E+10 -1.000000E+10 -1.000000E+10 -1.000000E+10 -1.000000E+10  0.000000E+00  0.000000E+00  0.000000E+00  0.000000E+00  0.000000E+00  0.000000E+00  0.000000E+00  0.000000E+00  0.000000E+00  0.000000E+00  0.000000E+00  0.000000E+00       Funtag_D
      82  3.861348E+05  3.473321E+05       38802.7       7.86098      0.103634       1.05678      0.187037      0.241399     0.0135393    -0.0103250    0.00803449       1.00886       Opttag_D
      82       82.8687       1.11258       1.00000  0.000000E+00       1.00000  0.000000E+00      0.945731       95.5160       1.00000       35.9648  0.000000E+00      0.667210  0.000000E+00      0.667210  0.000000E+00  0.000000E+00      -1.33442       82.1558      0.672497        1       Scatag_D
      82       1820.28       5558.06  0.000000E+00  0.000000E+00       2577.26  0.000000E+00       2266.81       7484.48  0.000000E+00       12659.7       46.7123       6389.40 -1.000000E+10 -1.000000E+10 -1.000000E+10 -1.000000E+10 -1.000000E+10  0.000000E+00  0.000000E+00  0.000000E+00  0.000000E+00  0.000000E+00  0.000000E+00  0.000000E+00  0.000000E+00  0.000000E+00  0.000000E+00  0.000000E+00  0.000000E+00       Funtag_D
      83  3.861067E+05  3.473009E+05       38805.8       6.90766      0.104798       1.06737      0.187011      0.241375     0.0136294    -0.0103684    0.00803354       1.00894       Opttag_D
      83       82.8979       1.11235       1.00000  0.000000E+00       1.00000  0.000000E+00      0.945757       95.4945       1.00000       35.9756  0.000000E+00      0.665125  0.000000E+00      0.665125  0.000000E+00  0.000000E+00      -1.33025       82.1558      0.672497        1       Scatag_D
      83       1819.85       5558.93  0.000000E+00  0.000000E+00       2578.71  0.000000E+00       2267.34       7481.09  0.000000E+00       12663.6       46.7078       6389.57 -1.000000E+10 -1.000000E+10 -1.000000E+10 -1.000000E+10 -1.000000E+10  0.000000E+00  0.000000E+00  0.000000E+00  0.000000E+00  0.000000E+00  0.000000E+00  0.000000E+00  0.000000E+00  0.000000E+00  0.000000E+00  0.000000E+00  0.000000E+00       Funtag_D
      84  3.860803E+05  3.472601E+05       38820.2       10.3503      0.106484       1.08247      0.186977      0.241355     0.0137471    -0.0104324    0.00803239       1.00912       Opttag_D
      84       82.9399       1.11218       1.00000  0.000000E+00       1.00000  0.000000E+00      0.945788       95.4705       1.00000       35.9925  0.000000E+00      0.662181  0.000000E+00      0.662181  0.000000E+00  0.000000E+00      -1.32436       82.1558      0.672497        1       Scatag_D
      84       1819.34       5560.98  0.000000E+00  0.000000E+00       2580.88  0.000000E+00       2268.42       7483.91  0.000000E+00       12669.7       46.5432       6390.38 -1.000000E+10 -1.000000E+10 -1.000000E+10 -1.000000E+10 -1.000000E+10  0.000000E+00  0.000000E+00  0.000000E+00  0.000000E+00  0.000000E+00  0.000000E+00  0.000000E+00  0.000000E+00  0.000000E+00  0.000000E+00  0.000000E+00  0.000000E+00       Funtag_D
      85  3.860525E+05  3.472233E+05       38829.2       7.65062      0.108051       1.09626      0.186947      0.241347     0.0138533    -0.0105134    0.00803265       1.00927       Opttag_D
      85       82.9782       1.11206       1.00000  0.000000E+00       1.00000  0.000000E+00      0.945816       95.4497       1.00000       36.0088  0.000000E+00      0.659629  0.000000E+00      0.659629  0.000000E+00  0.000000E+00      -1.31926       82.1558      0.672497        1       Scatag_D
      85       1819.45       5562.62  0.000000E+00  0.000000E+00       2582.99  0.000000E+00       2269.46       7481.04  0.000000E+00       12675.5       46.3457       6391.76 -1.000000E+10 -1.000000E+10 -1.000000E+10 -1.000000E+10 -1.000000E+10  0.000000E+00  0.000000E+00  0.000000E+00  0.000000E+00  0.000000E+00  0.000000E+00  0.000000E+00  0.000000E+00  0.000000E+00  0.000000E+00  0.000000E+00  0.000000E+00       Funtag_D
      86  3.860278E+05  3.471902E+05       38837.6       6.75878      0.109431       1.10823      0.186924      0.241362     0.0139488    -0.0106411    0.00803375       1.00941       Opttag_D
      86       83.0116       1.11191       1.00000  0.000000E+00       1.00000  0.000000E+00      0.945838       95.4336       1.00000       36.0249  0.000000E+00      0.657467  0.000000E+00      0.657467  0.000000E+00  0.000000E+00      -1.31493       82.1558      0.672497        1       Scatag_D
      86       1819.95       5564.17  0.000000E+00  0.000000E+00       2584.94  0.000000E+00       2270.16       7478.83  0.000000E+00       12680.7       46.1439       6392.72 -1.000000E+10 -1.000000E+10 -1.000000E+10 -1.000000E+10 -1.000000E+10  0.000000E+00  0.000000E+00  0.000000E+00  0.000000E+00  0.000000E+00  0.000000E+00  0.000000E+00  0.000000E+00  0.000000E+00  0.000000E+00  0.000000E+00  0.000000E+00       Funtag_D
      87  3.860028E+05  3.471350E+05       38867.8       9.93041      0.111833       1.12874      0.186882      0.241413     0.0141084    -0.0109155    0.00803670       1.00981       Opttag_D
      87       83.0691       1.11167       1.00000  0.000000E+00       1.00000  0.000000E+00      0.945878       95.4032       1.00000       36.0550  0.000000E+00      0.653767  0.000000E+00      0.653767  0.000000E+00  0.000000E+00      -1.30753       82.1558      0.672497        1       Scatag_D
      87       1821.28       5568.55  0.000000E+00  0.000000E+00       2588.42  0.000000E+00       2271.11       7487.71  0.000000E+00       12690.0       45.9329       6394.85 -1.000000E+10 -1.000000E+10 -1.000000E+10 -1.000000E+10 -1.000000E+10  0.000000E+00  0.000000E+00  0.000000E+00  0.000000E+00  0.000000E+00  0.000000E+00  0.000000E+00  0.000000E+00  0.000000E+00  0.000000E+00  0.000000E+00  0.000000E+00       Funtag_D
      88  3.859811E+05  3.470969E+05       38884.2       7.33551      0.113570       1.14329      0.186852      0.241459     0.0142184    -0.0111183    0.00804059       1.01007       Opttag_D
      88       83.1098       1.11155       1.00000  0.000000E+00       1.00000  0.000000E+00      0.945904       95.3824       1.00000       36.0777  0.000000E+00      0.651076  0.000000E+00      0.651076  0.000000E+00  0.000000E+00      -1.30215       82.1558      0.672497        1       Scatag_D
      88       1823.05       5571.39  0.000000E+00  0.000000E+00       2590.96  0.000000E+00       2271.64       7488.55  0.000000E+00       12696.6       45.8676       6396.18 -1.000000E+10 -1.000000E+10 -1.000000E+10 -1.000000E+10 -1.000000E+10  0.000000E+00  0.000000E+00  0.000000E+00  0.000000E+00  0.000000E+00  0.000000E+00  0.000000E+00  0.000000E+00  0.000000E+00  0.000000E+00  0.000000E+00  0.000000E+00       Funtag_D
      89  3.859638E+05  3.470809E+05       38882.9       5.93267      0.114155       1.14808      0.186840      0.241478     0.0142646    -0.0111961    0.00804084       1.01013       Opttag_D
      89       83.1230       1.11138       1.00000  0.000000E+00       1.00000  0.000000E+00      0.945915       95.3736       1.00000       36.0846  0.000000E+00      0.650070  0.000000E+00      0.650070  0.000000E+00  0.000000E+00      -1.30014       82.1558      0.672497        1       Scatag_D
      89       1823.17       5572.01  0.000000E+00  0.000000E+00       2591.91  0.000000E+00       2271.49       7484.67  0.000000E+00       12698.2       45.9314       6395.57 -1.000000E+10 -1.000000E+10 -1.000000E+10 -1.000000E+10 -1.000000E+10  0.000000E+00  0.000000E+00  0.000000E+00  0.000000E+00  0.000000E+00  0.000000E+00  0.000000E+00  0.000000E+00  0.000000E+00  0.000000E+00  0.000000E+00  0.000000E+00       Funtag_D
      90  3.859375E+05  3.470418E+05       38895.7       7.42049      0.115746       1.16098      0.186811      0.241509     0.0143774    -0.0113358    0.00804063       1.01042       Opttag_D
      90       83.1583       1.11105       1.00000  0.000000E+00       1.00000  0.000000E+00      0.945940       95.3519       1.00000       36.1023  0.000000E+00      0.647371  0.000000E+00      0.647371  0.000000E+00  0.000000E+00      -1.29474       82.1558      0.672497        1       Scatag_D
      90       1823.07       5575.21  0.000000E+00  0.000000E+00       2594.48  0.000000E+00       2271.60       7487.96  0.000000E+00       12702.9       46.0560       6394.37 -1.000000E+10 -1.000000E+10 -1.000000E+10 -1.000000E+10 -1.000000E+10  0.000000E+00  0.000000E+00  0.000000E+00  0.000000E+00  0.000000E+00  0.000000E+00  0.000000E+00  0.000000E+00  0.000000E+00  0.000000E+00  0.000000E+00  0.000000E+00       Funtag_D
      91  3.859254E+05  3.469890E+05       38936.4       14.6671      0.118298       1.18135      0.186771      0.241553     0.0145300    -0.0114831    0.00804468       1.01090       Opttag_D
      91       83.2141       1.11086       1.00000  0.000000E+00       1.00000  0.000000E+00      0.945975       95.3228       1.00000       36.1301  0.000000E+00      0.643547  0.000000E+00      0.643547  0.000000E+00  0.000000E+00      -1.28709       82.1558      0.672497        1       Scatag_D
      91       1824.91       5580.51  0.000000E+00  0.000000E+00       2598.46  0.000000E+00       2272.57       7508.72  0.000000E+00       12711.7       46.1209       6393.37 -1.000000E+10 -1.000000E+10 -1.000000E+10 -1.000000E+10 -1.000000E+10  0.000000E+00  0.000000E+00  0.000000E+00  0.000000E+00  0.000000E+00  0.000000E+00  0.000000E+00  0.000000E+00  0.000000E+00  0.000000E+00  0.000000E+00  0.000000E+00       Funtag_D
      92  3.858994E+05  3.469837E+05       38915.8       7.35371      0.118470       1.18264      0.186768      0.241546     0.0145454    -0.0114631    0.00804187       1.01083       Opttag_D
      92       83.2172       1.11076       1.00000  0.000000E+00       1.00000  0.000000E+00      0.945975       95.3221       1.00000       36.1302  0.000000E+00      0.643248  0.000000E+00      0.643248  0.000000E+00  0.000000E+00      -1.28650       82.1558      0.672497        1       Scatag_D
      92       1823.63       5579.77  0.000000E+00  0.000000E+00       2598.78  0.000000E+00       2272.76       7490.18  0.000000E+00       12712.0       46.0774       6392.58 -1.000000E+10 -1.000000E+10 -1.000000E+10 -1.000000E+10 -1.000000E+10  0.000000E+00  0.000000E+00  0.000000E+00  0.000000E+00  0.000000E+00  0.000000E+00  0.000000E+00  0.000000E+00  0.000000E+00  0.000000E+00  0.000000E+00  0.000000E+00       Funtag_D
      93  3.858747E+05  3.469604E+05       38914.3       6.22655      0.119551       1.19101      0.186750      0.241542     0.0146126    -0.0114608    0.00803974       1.01096       Opttag_D
      93       83.2394       1.11060       1.00000  0.000000E+00       1.00000  0.000000E+00      0.945982       95.3144       1.00000       36.1388  0.000000E+00      0.641744  0.000000E+00      0.641744  0.000000E+00  0.000000E+00      -1.28349       82.1558      0.672497        1       Scatag_D
      93       1822.66       5581.23  0.000000E+00  0.000000E+00       2600.54  0.000000E+00       2273.61       7483.65  0.000000E+00       12715.5       45.9629       6391.21 -1.000000E+10 -1.000000E+10 -1.000000E+10 -1.000000E+10 -1.000000E+10  0.000000E+00  0.000000E+00  0.000000E+00  0.000000E+00  0.000000E+00  0.000000E+00  0.000000E+00  0.000000E+00  0.000000E+00  0.000000E+00  0.000000E+00  0.000000E+00       Funtag_D
      94  3.858517E+05  3.469236E+05       38928.1       7.32244      0.121383       1.20506      0.186723      0.241549     0.0147188    -0.0115032    0.00803986       1.01123       Opttag_D
      94       83.2769       1.11041       1.00000  0.000000E+00       1.00000  0.000000E+00      0.945996       95.3009       1.00000       36.1557  0.000000E+00      0.639354  0.000000E+00      0.639354  0.000000E+00  0.000000E+00      -1.27871       82.1558      0.672497        1       Scatag_D
      94       1822.72       5584.17  0.000000E+00  0.000000E+00       2603.46  0.000000E+00       2274.92       7485.49  0.000000E+00       12721.7       45.8263       6389.83 -1.000000E+10 -1.000000E+10 -1.000000E+10 -1.000000E+10 -1.000000E+10  0.000000E+00  0.000000E+00  0.000000E+00  0.000000E+00  0.000000E+00  0.000000E+00  0.000000E+00  0.000000E+00  0.000000E+00  0.000000E+00  0.000000E+00  0.000000E+00       Funtag_D
      95  3.858225E+05  3.468575E+05       38965.0       14.8233      0.124675       1.22947      0.186676      0.241559     0.0149096    -0.0115671    0.00803926       1.01177       Opttag_D
      95       83.3415       1.10997       1.00000  0.000000E+00       1.00000  0.000000E+00      0.946012       95.2794       1.00000       36.1844  0.000000E+00      0.635566  0.000000E+00      0.635566  0.000000E+00  0.000000E+00      -1.27113       82.1558      0.672497        1       Scatag_D
      95       1822.45       5590.20  0.000000E+00  0.000000E+00       2608.80  0.000000E+00       2277.56       7501.30  0.000000E+00       12732.0       45.5542       6387.15 -1.000000E+10 -1.000000E+10 -1.000000E+10 -1.000000E+10 -1.000000E+10  0.000000E+00  0.000000E+00  0.000000E+00  0.000000E+00  0.000000E+00  0.000000E+00  0.000000E+00  0.000000E+00  0.000000E+00  0.000000E+00  0.000000E+00  0.000000E+00       Funtag_D
      96  3.857911E+05  3.468139E+05       38977.2       7.20172      0.127452       1.24970      0.186643      0.241570     0.0150356    -0.0116074    0.00804118       1.01206       Opttag_D
      96       83.3959       1.10997       1.00000  0.000000E+00       1.00000  0.000000E+00      0.946023       95.2631       1.00000       36.2123  0.000000E+00      0.632493  0.000000E+00      0.632493  0.000000E+00  0.000000E+00      -1.26499       82.1558      0.672497        1       Scatag_D
      96       1823.32       5593.38  0.000000E+00  0.000000E+00       2613.15  0.000000E+00       2279.85       7493.69  0.000000E+00       12742.3       45.4189       6386.07 -1.000000E+10 -1.000000E+10 -1.000000E+10 -1.000000E+10 -1.000000E+10  0.000000E+00  0.000000E+00  0.000000E+00  0.000000E+00  0.000000E+00  0.000000E+00  0.000000E+00  0.000000E+00  0.000000E+00  0.000000E+00  0.000000E+00  0.000000E+00       Funtag_D
      97  3.857740E+05  3.468089E+05       38965.1       5.58525      0.127448       1.24934      0.186639      0.241560     0.0150500    -0.0115734    0.00803809       1.01190       Opttag_D
      97       83.3944       1.10981       1.00000  0.000000E+00       1.00000  0.000000E+00      0.946020       95.2634       1.00000       36.2082  0.000000E+00      0.632630  0.000000E+00      0.632630  0.000000E+00  0.000000E+00      -1.26526       82.1558      0.672497        1       Scatag_D
      97       1821.91       5591.64  0.000000E+00  0.000000E+00       2613.24  0.000000E+00       2279.68       7487.31  0.000000E+00       12740.9       45.4696       6384.97 -1.000000E+10 -1.000000E+10 -1.000000E+10 -1.000000E+10 -1.000000E+10  0.000000E+00  0.000000E+00  0.000000E+00  0.000000E+00  0.000000E+00  0.000000E+00  0.000000E+00  0.000000E+00  0.000000E+00  0.000000E+00  0.000000E+00  0.000000E+00       Funtag_D
      98  3.857411E+05  3.467825E+05       38958.6       8.18039      0.128274       1.25413      0.186617      0.241523     0.0151262    -0.0114732    0.00802976       1.01168       Opttag_D
      98       83.4053       1.10932       1.00000  0.000000E+00       1.00000  0.000000E+00      0.946014       95.2585       1.00000       36.2030  0.000000E+00      0.632206  0.000000E+00      0.632206  0.000000E+00  0.000000E+00      -1.26441       82.1558      0.672497        1       Scatag_D
      98       1818.14       5589.23  0.000000E+00  0.000000E+00       2614.88  0.000000E+00       2280.01       7488.87  0.000000E+00       12740.3       45.6210       6381.53 -1.000000E+10 -1.000000E+10 -1.000000E+10 -1.000000E+10 -1.000000E+10  0.000000E+00  0.000000E+00  0.000000E+00  0.000000E+00  0.000000E+00  0.000000E+00  0.000000E+00  0.000000E+00  0.000000E+00  0.000000E+00  0.000000E+00  0.000000E+00       Funtag_D
      99  3.857223E+05  3.467409E+05       38981.5       15.1594      0.130259       1.26672      0.186583      0.241470     0.0152465    -0.0113407    0.00802329       1.01166       Opttag_D
      99       83.4369       1.10885       1.00000  0.000000E+00       1.00000  0.000000E+00      0.946010       95.2467       1.00000       36.2060  0.000000E+00      0.630724  0.000000E+00      0.630724  0.000000E+00  0.000000E+00      -1.26145       82.1558      0.672497        1       Scatag_D
      99       1815.21       5588.91  0.000000E+00  0.000000E+00       2618.43  0.000000E+00       2281.47       7509.18  0.000000E+00       12744.8       45.7691       6377.64 -1.000000E+10 -1.000000E+10 -1.000000E+10 -1.000000E+10 -1.000000E+10  0.000000E+00  0.000000E+00  0.000000E+00  0.000000E+00  0.000000E+00  0.000000E+00  0.000000E+00  0.000000E+00  0.000000E+00  0.000000E+00  0.000000E+00  0.000000E+00       Funtag_D

   MaxLik Scaling Cycle 0104

   Wilson plot

   Statistics in resolution bins after refinement cycle 0003
```


|  |  |
| --- | --- |
|  | - Rxpct factors plot- Rxpct factors table- <Log-Lik Gain>- Ln Sigma A- Correlation Coefficients |


```
   Iters     Tot_Funct    Xray_Funct    Geom_Funct          Grms     rmsD2init     maxD2init             R         Rfree           LLG       LLGfree     rms(bond)    rms(angle)
   Iters             K   B_IMPF_FRAG        K_MISS   B_TEMP_MISS   K_IMPF_MISS   B_IMPF_MISS        K_SOLV   B_TEMP_SOLV   K_IMPF_SOLV   B_IMPF_SOLV        B_TEMP     B_TEMP_11     B_TEMP_12     B_TEMP_22     B_TEMP_13     B_TEMP_23     B_TEMP_33      K_WILSON      B_WILSON      FGCYCLES
   Iters          BOND         ANGLE      IMPROPER       TORSION    SINTORSION      TRIGONAL        PLANAR  BCORRELATION       CONTACT     IDEALDIST        CHIRAL    SIMILARITY       OCCSSUM  UTILDISTANCE     UTILANGLE   UTILTORSION        RHOFIT        ADP-01        ADP-02        ADP-03        ADP-04        ADP-05        ADP-06        ADP-07        ADP-08        ADP-09        ADP-10      RHOFITPC            QM
     100  3.856925E+05  3.467300E+05       38962.6       9.01555      0.130829       1.27021      0.186574      0.241449     0.0152780    -0.0112987    0.00801981       1.01160       Opttag_D
     100       83.4454       1.10873       1.00000  0.000000E+00       1.00000  0.000000E+00      0.946008       95.2437       1.00000       36.2054  0.000000E+00      0.630441  0.000000E+00      0.630441  0.000000E+00  0.000000E+00      -1.26088       82.1558      0.672497        1       Scatag_D
     100       1813.64       5588.32  0.000000E+00  0.000000E+00       2619.45  0.000000E+00       2281.88       7491.05  0.000000E+00       12746.3       45.7844       6376.09 -1.000000E+10 -1.000000E+10 -1.000000E+10 -1.000000E+10 -1.000000E+10  0.000000E+00  0.000000E+00  0.000000E+00  0.000000E+00  0.000000E+00  0.000000E+00  0.000000E+00  0.000000E+00  0.000000E+00  0.000000E+00  0.000000E+00  0.000000E+00       Funtag_D
   Geometry Restraint Deviations SCREEN Information

 
 No -bcorrel_zeroocchydrogens_factor flag found - keeping default: will set B for OCC=0 hydrogens to    1.200 times heavy-atom B


 TLS information. Total number of groups:     2


 Group   Natoms    FixRB   FixTLS   Group Tag    Group Spec
     1     2783      Y        Y     tls1    { A|* }
     2     2770      Y        Y     tls2    { B|* }
 
NOTE BUSTER_TLS_SET tls1 { A|* }
NOTE BUSTER_TLS_T   tls1     -2.34221E-01 -1.01327E-01  3.03964E-01 -4.69383E-02  3.04493E-02 -1.84208E-02
NOTE BUSTER_TLS_L   tls1      1.34657E+00  1.70940E+00  9.47056E-01 -1.47356E-01 -1.08231E+00  4.33010E-01
NOTE BUSTER_TLS_S   tls1      1.90995E-01 -3.20182E-01  2.99606E-01 -2.04932E-01  7.24381E-02 -2.58989E-01 -5.15178E-02 -2.36847E-01
NOTE BUSTER_TLS_O   tls1     -2.31731E+01 -8.40215E+01  1.10770E+01
NOTE BUSTER_TLS_FIX tls1 ALL
 
NOTE BUSTER_TLS_SET tls2 { B|* }
NOTE BUSTER_TLS_T   tls2     -2.94169E-01 -2.16545E-01  3.03964E-01  2.06527E-02  3.69318E-02 -2.62022E-02
NOTE BUSTER_TLS_L   tls2      1.48295E+00  1.25715E+00  8.55781E-01  5.77497E-01 -7.83848E-01 -7.57220E-01
NOTE BUSTER_TLS_S   tls2      1.16036E-02  8.23206E-02 -1.48910E-01  5.36303E-02  1.62509E-01  2.17161E-02 -7.83320E-02  1.02175E-01
NOTE BUSTER_TLS_O   tls2      2.22040E+01 -7.22942E+01  3.58164E+00
NOTE BUSTER_TLS_FIX tls2 ALL

 For final position, Geometry function summary
     term                           number     weight       rms        GooF      function     fn/numb
     ================================================================================================
     BOND bond lengths (angs)       5668       2.000      0.0080       0.400      1813.64       0.320
     ANGL bond angles (degs)        7691       2.000        1.01       0.603      5588.32       0.727
     TORS split-harmonic tors       1275       0.000       18.62       1.170         0.00       0.000
     SINTOR sinusoidal tors         1275       2.000       18.62                  2619.45       2.054
     PLAN general plane (angs)       967       5.000      0.0137       0.687      2281.88       2.360
     BCORrelations (angs^2)         5668      10.000       1.018       0.364      7491.05       1.322
     CONT (bad contacts)               0       5.000                                 0.00
     IDEAL(ideal-dist contact)      5586       4.000                             12746.34
     CHIRAL (gelly semiharmon)       740       5.000                                45.78
     SIM similarity restraint for NCS and/or TARGET                               6376.09
     OCCSUM restraint on sum of occupanies                                           0.00       as none defined
     DISTAN utility restraints interatomic distance                                  0.00       as none defined
     UTILANGLE utility angle restraints                                              0.00       as none defined
     UTILTOR utility restraints on torsion angles                                    0.00       as none defined
     Peptide omega torsion angles                           2.83 degs - monitor only
 
 WARNING: Have |delta/sigma| deviations > 5.0 sigma. Number of outliers for each term: 
 WARNING:         2 bond angles.  Worst is     5.1 sigs   134.54 degs A|28:C.A(ASN)=A|29:N(ASN)=A|29:CA(ASN)

 Weighted rms      0.0080         1.012  (temporary rms bond and angle for BUSTER to pick up!)
 Weighted intra-residual rms(bond) for StdProtein (excluding H)      0.0078
 Weighted intra-residual rms(bond) for Buffer     (excluding H)      0.0030
 Outputing geometry progress graph in plotmtv format to file:Geom_Res.mtv

 ############### Running final geometry/screen analysis (screen_final.txt)

 s/r gelly normal termination
```

Statistics and scale factors vs. cycle number
```

|  |  |  |
| --- | --- | --- |
|  | - Rxpct factors- <Log-Lik Gain>- GEOM Residuals- Ln Sigma_A_ | - Scale Factors- B Factors- Imperfection Factors |

```
   Final Geometry Information


---


### 2. Final results

Statistics and scale factors vs. cycle number
```

|  |  |  |
| --- | --- | --- |
|  | - Rxpct factors- <Log-Lik Gain>- GEOM Residuals- Ln Sigma_A_ | - Scale Factors- B Factors- Imperfection Factors |

```
   Average Figure of Merit
   Structure Factor Amplitudes


 # normal termination

  Program stopped.
```

closesok message: time spent in tree = 1736.52 (user) 24.55 (sys)

---

# Normal termination

```
 Resources usage summary:
     Time spent in client ...................  1736.53 (user)       24.56 (system)
     Time spent in scripts (    27 calls) ...     2.20 (user)        0.14 (system)
     Time spent in server ...................     0.01 (user)        0.00 (system)

     Max resident memory used (kb) .......... 378212
     Number of page faults .................. 0 (major)    406418 (minor)
     Number of context switches ............. 3673062 (niced)    9712 (other)
```
